# Increased fatty acid delivery by tumor endothelium promotes metastatic outgrowth

**DOI:** 10.1172/jci.insight.187531

**Published:** 2025-04-08

**Authors:** Deanna N. Edwards, Shan Wang, Kelby Kane, Wenqiang Song, Laura C. Kim, Verra M. Ngwa, Yoonha Hwang, Kevin Ess, Mark R. Boothby, Jin Chen

**Affiliations:** 1 Department of Medicine, Division of Rheumatology and Immunology, Vanderbilt University Medical Center, Nashville, Tennessee, USA.; 2 Vanderbilt-Ingram Cancer Center, Nashville, Tennessee, USA.; 3 Program in Cancer Biology, Vanderbilt University, Nashville, Tennessee, USA.; 4 Department of Medicine, Division of Epidemiology, and; 5 Department of Pathology, Microbiology and Immunology, Vanderbilt University Medical Center, Nashville, Tennessee, USA.; 6 Abramson Family Cancer Research Institute, Perelman School of Medicine, University of Pennsylvania, Philadelphia, Pennsylvania, USA.; 7 Department of Pediatrics, University of Colorado Anschutz Medical Campus, Denver, Colorado, USA.; 8 Department of Pediatrics, Vanderbilt University Medical Center, Nashville, Tennessee, USA.; 9 Vanderbilt Institute for Infection, Immunology, and Inflammation, Nashville, Tennessee, USA.; 10 Department of Cell and Developmental Biology, Vanderbilt University, Nashville, Tennessee, USA.; 11 Veterans Affairs Medical Center, Tennessee Valley Healthcare System, Nashville, Tennessee, USA.

**Keywords:** Immunology, Metabolism, Oncology, Cancer immunotherapy, Endothelial cells

## Abstract

Metastatic outgrowth in distant microscopic niches requires sufficient nutrients, including fatty acids (FAs), to support tumor growth and to generate an immunosuppressive tumor microenvironment (TME). However, despite the important role of FAs in metastasis, the regulation of FA supply in metastatic niches has not been defined. In this report, we show that tumor endothelium actively promotes outgrowth and restricts antitumor cytolysis by transferring FAs into developing metastatic tumors. We describe a process of transendothelial FA delivery via endosomes that requires mTORC1 activity. Thus, endothelial cell–specific targeted deletion of Raptor (*Rptor*^ECKO^), a unique component of the mTORC1 complex, significantly reduced metastatic tumor burden that was associated with improved markers of T cell cytotoxicity. Low-dose everolimus that selectively inhibited endothelial mTORC1 improves immune checkpoint responses in metastatic disease models. This work reveals the importance of transendothelial nutrient delivery to the TME, highlighting a future target for therapeutic development.

## Abstract 2

Tumor endothelial cells promote delivery of fatty acids into developing metastatic tumors, creating an immunosuppressive microenvironment that supports outgrowth of disseminated cancer cells.

## Graphical abstract

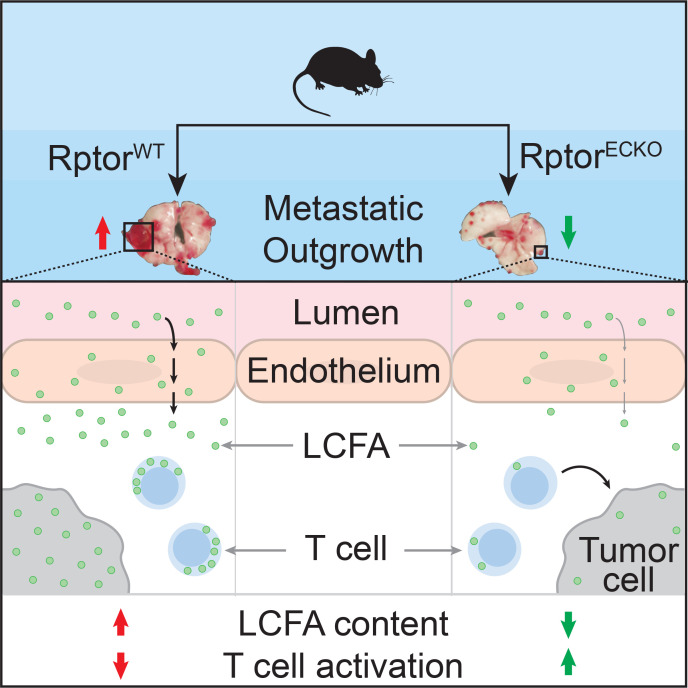

## Introduction

Proliferative outgrowth of metastatic tumor cells requires sufficient nutrient availability to support energetics and biomass production (1). Although the exact metabolic requirements for metastasis have not been fully defined, recent evidence suggests that fatty acid uptake is necessary for metastatic progression (2–4). Compared with normal lung or primary mammary tumors, the interstitial fluid of lung metastatic tumors has drastically elevated levels of long-chain fatty acids (LCFAs) such as palmitate, serving to support the high metabolic demands of disseminated tumor cells (3). Other studies have indicated that fatty acid–enriched environments suppress cytotoxic T lymphocyte (CTL) activity (5, 6), capable of further supporting metastatic outgrowth. However, there is no indication of how fatty acid delivery is regulated in the early lung metastatic niche.

The vascular system is well recognized as a critical distributor of oxygen and nutrients. Endothelial cells lining vessel walls have been described to deliver free fatty acids from the blood to surrounding tissues via a transendothelial delivery mechanism (7, 8). The process is dependent on vascular endothelial growth factor B (VEGF-B) signaling through VEGF receptor 1 (VEGFR1) and most prominently occurs in adipose tissue, heart, and skeletal muscle (7, 8). Like these highly metabolic tissues, tumors are excessive producers of VEGF-B (9–11), suggesting that tumor vasculature may support delivery of fatty acids to tumor cells. In contrast with VEGF-A, VEGF-B does not appear to markedly impact angiogenesis (12–14), but a recent study demonstrated that it supports spontaneous metastatic colonization in the lung (11). Due to the highly vascularized nature of the lung, transendothelial delivery of fatty acids may be particularly important to support the elevated metabolic demands of tumor cells during metastatic outgrowth, which warrants further investigation.

The serine/threonine kinase mTOR is a common signaling node downstream of vascular growth factor signaling (15, 16). The mTOR kinase forms 2 functionally distinct complexes with shared (e.g., mTOR and mLST8) and unique components (e.g., regulatory-associated protein of mammalian target of rapamycin [Raptor] in mTORC1 and rapamycin independent companion of target of rapamycin [Rictor] in mTORC2). mTORC1 integrates environmental cues with regulation of important cellular processes, including metabolism, proliferation, and survival (15, 16). TSC2, a component of the TSC complex, through its GTPase-activating domain inactivates Rheb to inhibit mTORC1 activity (17, 18). Aberrant mTORC1 activity in endothelial cells has been attributed to tumor development (19–21), but its role within the early metastatic lung niche remains to be determined.

In this report, we demonstrate that endothelial cell–specific deletion of Raptor (*Rptor*^ECKO^) reduces lung metastatic outgrowth. Loss of Raptor in endothelial cells reduces intracellular LCFA uptake and transendothelial delivery of a fluorescent palmitate analog (BODIPY-C16) to metastatic tumor cells and T lymphocytes. In contrast, loss of endothelial cell TSC2 (*Tsc2*^ECKO^) increased metastatic outgrowth and transendothelial delivery of LCFAs. We demonstrate that Raptor-deficient endothelial cells exhibit deficiencies in endosomal trafficking, having reduced expression of calysintenin-1 (*Clstn1*) involved in anterograde endosomal transport that correlates with an mTORC1 activity gene signature in human tumor–associated endothelial cells. Indeed, knockdown of *Clstn1* reduced transendothelial transport of BODIPY-C16 in control but not Raptor-deficient endothelial cells. Reduced delivery of LCFAs to *Rptor*^ECKO^ metastatic tumors enhanced antitumor immune responses of T cells, suggesting that selective targeting of endothelial mTORC1 may improve the tumor microenvironment (TME) during metastatic outgrowth. Using a low dose of the mTORC1 inhibitor everolimus (RAD001) in combination with an anti–PD-1 (αPD-1) antibody reduced BODIPY-C16 uptake in metastatic tumor cells and T cells, improving T cell responses. Taken together, our findings suggest that Raptor/mTORC1 supports transendothelial delivery of LCFAs to support metastatic outgrowth.

## Results

### Endothelial mTORC1 promotes metastatic outgrowth in the lung.

**Figure 1:**
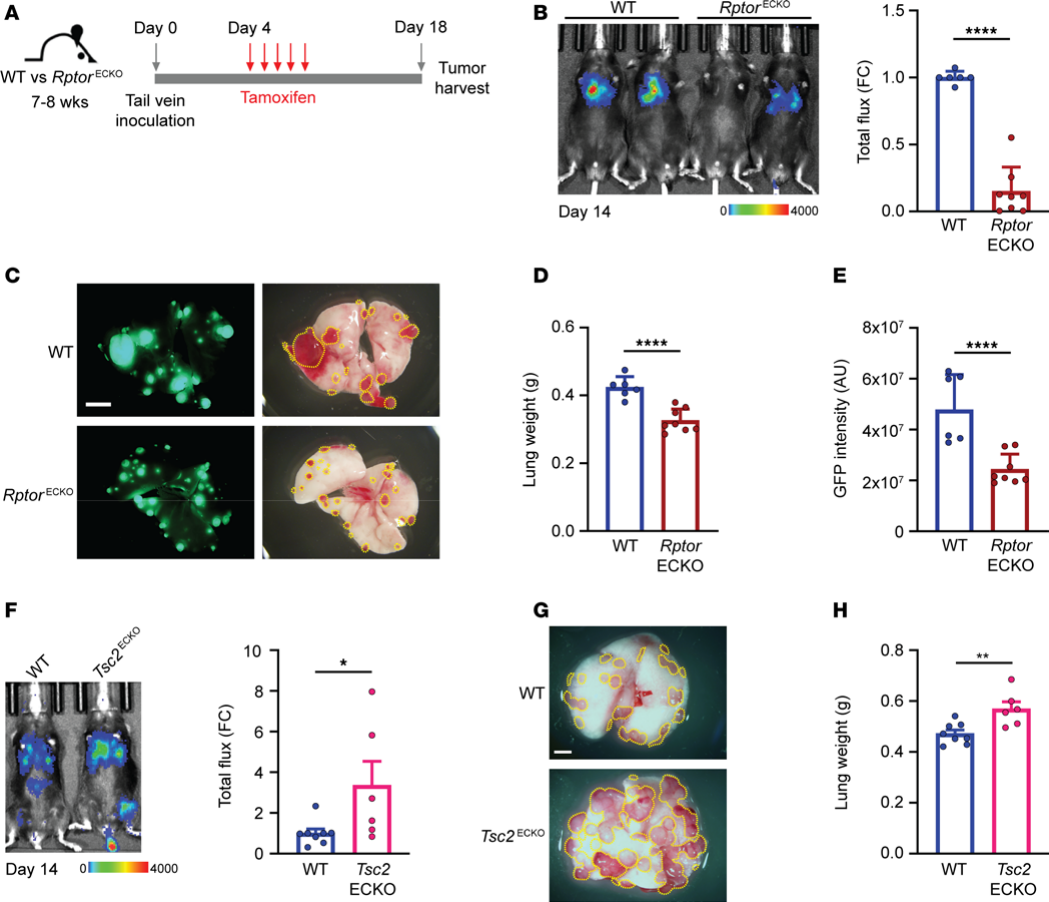
Raptor/mTORC1 loss in endothelium reduces metastatic outgrowth in the lung. (**A**) Schematic of experimental procedures of tumor cell inoculation, tamoxifen treatment, and harvest. (**B**–**E**) WT (*n* = 6) or *Rptor*^ECKO^ (*n* = 8) male mice were inoculated with LLC-GFP-luc cells and treated with tamoxifen, as described in **A**. (**B**) Representative bioluminescence image from day 14 is shown. Scale bar shows counts. Total radiance flux was normalized to WT controls and presented as fold change (FC). (**C**) Representative GFP (left) and gross (right) lungs after harvest on day 18. Scale bar: 5 mm. Visible tumor area is outlined by yellow line. (**D**) Lung weights were recorded in grams (g) at harvest and (**E**) GFP intensity was calculated as arbitrary units (a.u.). (**F**–**H**) WT (*n* = 8) or *Tsc2*^ECKO^ (*n* = 6) female mice were inoculated with E0771-luc tumor cells as described in **A**. (**F**) A representative bioluminescence image is shown from day 14. Scale bar shows counts. Total radiance flux was normalized to WT controls. (**G**) Representative lungs after harvest on day 20 are shown. Scale bar: 5 mm. Visible tumor area is outlined by yellow line. (**H**) Lung weights were recorded in grams (g) at harvest. *P < 0.05, **P < 0.01, ***P < 0.005, ****P < 0.001 by unpaired, 2-tailed Student’s *t* test.

To evaluate how endothelial mTORC1 impacts metastatic outgrowth in the lung, we used an inducible genetically engineered mouse model to selectively delete Raptor in endothelial cells (21). Mice carrying floxed *Rptor* alleles (*Rptor^fl/fl^*, referred to as WT) were crossed with mice harboring a tamoxifen-inducible Cre recombinase (Cre^ER^) under the control of the vascular endothelial cadherin (*Cdh5*/VE-Cad) promoter (referred to as *Rptor*^ECKO^). Loss of Raptor reduced phosphorylation of S6 (p-S6) ribosomal protein, a marker of mTORC1 signaling (Supplemental Figure 1A; supplemental material available online with this article; https://doi.org/10.1172/jci.insight.187531DS1). However, loss of mTORC1 in adult endothelial cells does not affect quiescent vasculature in healthy adult animals (21). To model metastatic outgrowth, we intravenously injected murine Lewis lung carcinoma (LLC) cells engineered to express GFP and luciferase (LLC-GFP-luc) (Figure 1A). To allow even seeding in the lung niche that is not influenced by endothelial Raptor status, inoculation occurred prior to tamoxifen administration and Cre induction (Supplemental Figure 1B). Raptor loss in endothelial cells (*Rptor*^ECKO^) significantly reduced LLC lung tumor burden based on bioluminescence imaging, lung weights, and GFP intensity (Figure 1, B–E). Similar results were obtained following intravenous inoculation of MMTV-PyMT (Supplemental Figure 1, C and D) and E0771 (Supplemental Figure 1, E–H) murine breast cancer models.

To rigorously examine the role of endothelial mTORC1 in metastatic outgrowth, we also generated mice lacking endothelial TSC2 (*Tsc2*^ECKO^) (Supplemental Figure 1I), a key negative regulator of mTORC1, to model increased mTORC1 activity in endothelial cells. *Tsc2*^ECKO^ animals developed greater tumor burden compared with controls (Figure 1, F–H), consistent with a role of endothelial mTORC1 in promoting metastatic outgrowth in the lung. However, targeting endothelial Rictor (*Rictor*^ECKO^), a unique and required component of mTORC2, had no significant impact on lung tumor burden (Supplemental Figure 2), indicating the critical role of endothelial mTORC1, but not mTORC2, on metastatic outgrowth in the lung.

### Endothelial mTORC1 increases LCFA uptake in endothelial cells downstream of VEGF-B/VEGFR1.

**Figure 2:**
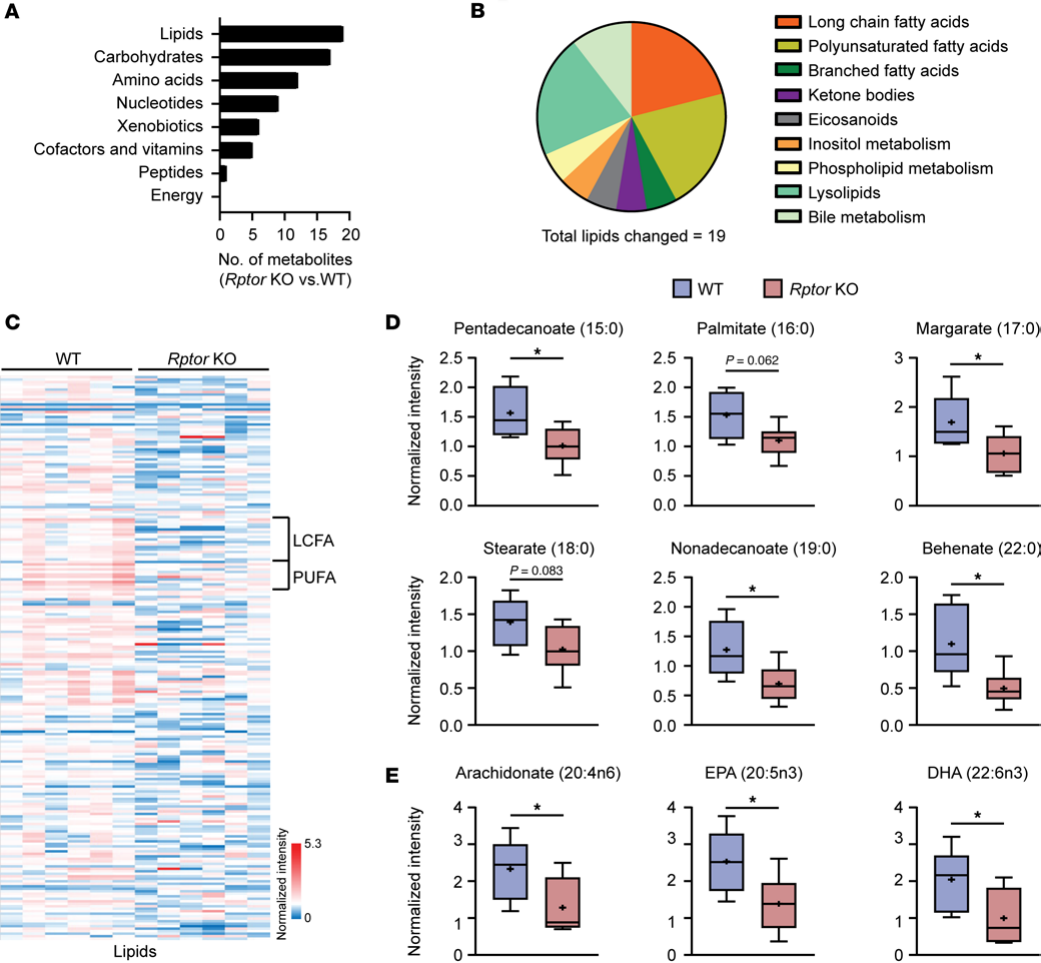
Long-chain fatty acids are reduced upon loss of Raptor/mTORC1 in endothelial cells. Metabolomics was performed on primary microvascular endothelial cells isolated from *Rptor^fl/fl^* mice transduced with control (WT) or Cre-recombinase (*Rptor*-KO) adenoviruses. Cells were collected 24 hours after infection (*n* = 6 per group). (**A** and **B**) Summary of significantly altered metabolites by (**A**) major class and (**B**) lipid classes in *Rptor*-KO versus WT endothelial cells. (**C**) Heatmap of lipid metabolites in WT and *Rptor*-KO endothelial cells. Columns represent individual samples and rows are metabolites. Long-chain fatty acids (LCFAs) and long-chain polyunsaturated fatty acids (PUFAs) are indicated. (**D** and **E**) Normalized intensities (log_2_ + 1) of representative (**D**) LCFA and (**E**) PUFA metabolites. The median along with the 25th and 75th percentile hinges are indicated within the box. The whiskers indicate minimum and maximum values within each group. The mean is shown as a plus sign (“+”). *P < 0.05 by 2-tailed Welch’s *t* test.

Due to the critical role of mTORC1 in regulating metabolism, we evaluated how mTORC1 status impacted the metabolome of control and Raptor-knockout (*Rptor*-KO) primary murine lung microvascular endothelial cells. Lipid metabolites were the most frequently altered by *Rptor* loss (Figure 2A, Supplemental Figure 3A, and Supplemental Table 1). Specifically, LCFA and long-chain polyunsaturated fatty acid content was collectively reduced in *Rptor*-KO endothelial cells (Figure 2, B–E), while shorter-chain fatty acid and medium-chain fatty acid (MCFA) content was unaffected (Supplemental Figure 3B).

The reduction in LCFA content within *Rptor*-KO endothelial cells suggests that mTORC1 regulates intracellular fatty acid levels. Through VEGFR1, VEGF-B was previously reported to increase fatty acid uptake by endothelial cells (7), although the signaling mechanism was not defined. Therefore, we examined whether mTORC1 increases fatty acid levels downstream of VEGF-B and VEGFR1 in primary lung murine microvascular endothelial cells. Loss of Raptor decreased expression of the fatty acid transporters and binding proteins in endothelial cells (Supplemental Figure 3, C–E), suggesting that fatty acid uptake and transport may be impaired. VEGF-B significantly increased the intracellular content of BODIPY-C16, a fluorescent palmitate analog, in control but not *Rptor*-KO endothelial cells (Supplemental Figure 3, F–H). Addition of a VEGFR1-neutralizing antibody diminished these effects of VEGF-B in control cells, while VEGF-A did not enhance BODIPY-C16 uptake, indicating that VEGF-B regulates LCFA uptake via VEGFR1 (Supplemental Figure 3, G and H). In contrast, neither VEGF-B nor Raptor status had any impact on intracellular levels of BODIPY-C12, a lauric acid (MCFA) fluorescent analog (Supplemental Figure 3, I–K). Together, these results indicate that VEGF-B/VEGFR1 and mTORC1 specifically promote LCFA uptake in endothelial cells.

Given that VEGF-B promotes LCFA uptake in a Raptor/mTORC1-dependent manner, we evaluated whether VEGF-B promotes mTORC1 activity in endothelial cells under serum starvation conditions. Western blot analysis showed that VEGF-B moderately increased phosphorylation of downstream mTORC1 targets, including S6K1, S6RP, and 4EBP1, but it had no significant impact on phosphorylation of the mTORC2 target AKT (Supplemental Figure 4, A and B). Not surprisingly, mTORC1 activity was not impacted by VEGF-B in *Rptor*-KO endothelial cells (Supplemental Figure 4, A and B). Using a more quantitative method, flow cytometry revealed that a VEGFR1-neutralizing antibody significantly reduced p-S6 levels in tumor-associated endothelial cells in vivo (Supplemental Figure 4C). Overall, these findings indicate that VEGF-B and VEGFR1 function upstream of mTORC1 to augment LCFA uptake in endothelial cells.

### Endothelial mTORC1 promotes transendothelial delivery of LCFAs.

**Figure 3:**
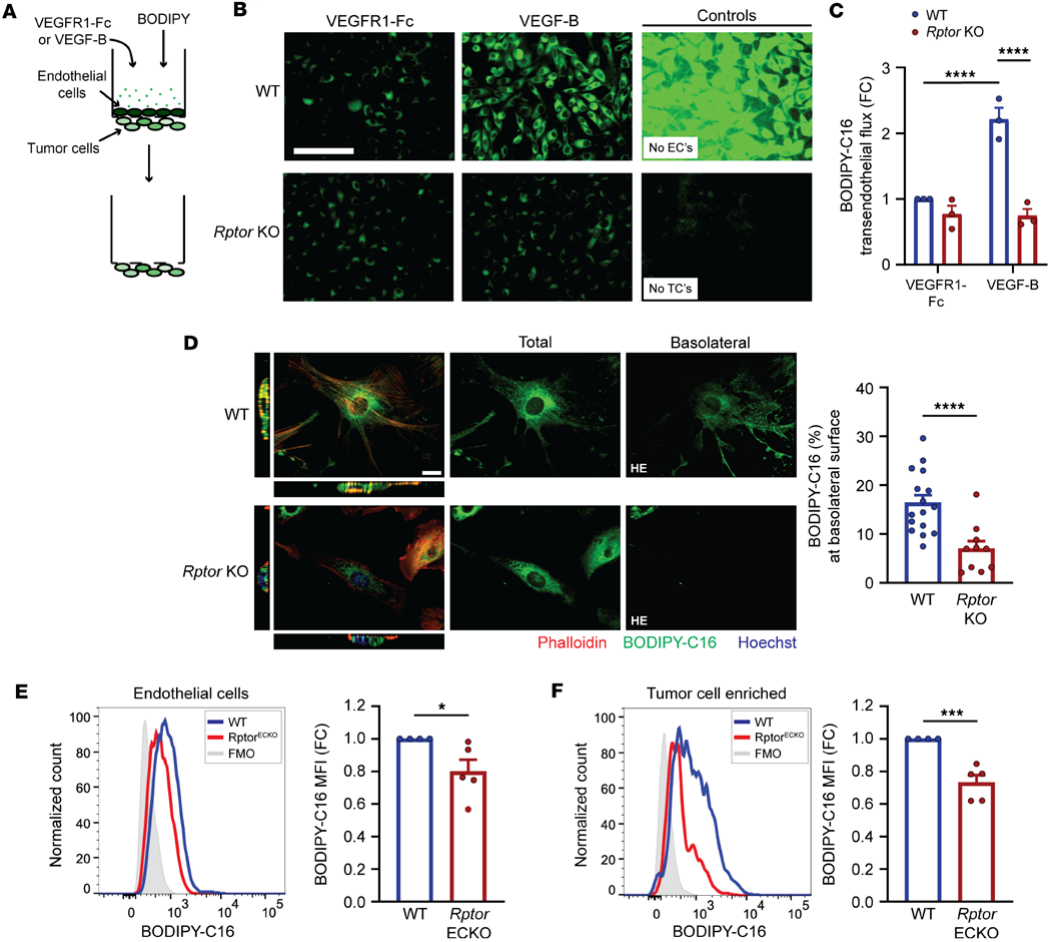
Endothelial Raptor/mTORC1 supports transendothelial delivery of fatty acids. (**A**) Schematic of transendothelial transport assay. (**B**) Representative images of BODIPY-C16 (green) in LLC tumor cells after 1 hour. VEGFR1-Fc was used in control samples to bind tumor cell–derived VEGF-B. Endothelial cells act as a physical barrier for BODIPY-C16 access, demonstrated in a representative image from an endothelial cell–free control (“No ECs”). A control performed without tumor cells (“No TCs”) is shown to confirm removal of endothelial cells prior to fluorescence imaging of basolateral tumor cells. Scale bar: 100 μm. (**C**) BODIPY-C16 fluorescence in LLC tumor cells, normalized to WT plus VEGFR1-Fc control (*n* = 3 per group). (**D**) Representative confocal images of WT or *Rptor*-KO endothelial cells treated with BODIPY-C16 for 5 minutes. Nuclear (Hoechst, blue) and actin (phalloidin, red) staining was used to detect perinuclear and cell boundaries, respectively. Total (all *z*-planes) and basolateral (bottom 10% of *z*-planes) BODIPY-C16 (green) staining are also shown, with the basolateral BODIPY presented at higher exposure (HE). Scale bar: 20 μm. BODIPY-C16 intensities at the basolateral surface were normalized to total signal in each cell. WT (*n* = 16) and *Rptor*-KO (*n* = 10) cells were analyzed from 2 independent experiments. (**E** and **F**) WT (*n* = 4) or *Rptor*^ECKO^ (*n* = 5) mice were inoculated with LLC tumor cells as described in Figure 1A. One hour prior to tumor harvest, animals were injected with BODIPY-C16 (50 μg). Median fluorescence intensity (MFI) was determined by flow cytometry in (**E**) CD45^–^CD31^+^ endothelial cells and (**F**) CD45^–^EpCAM^+^FSC^hi^ tumor cell–enriched populations and normalized to littermate WT controls. *P < 0.05, **P < 0.01, ***P < 0.005, ****P < 0.001 by 2-way ANOVA with Tukey’s post hoc test (**B**) or unpaired, 2-tailed Student’s *t* test (**D**–**F**).

The elevated fatty acid uptake associated with VEGF-B supports transendothelial delivery of fatty acids to tissues (7). To examine whether Raptor/mTORC1 promotes transendothelial LCFA delivery in response to VEGF-B, we utilized a Transwell assay to measure BODIPY-C16 movement across a confluent endothelial cell layer toward tumor cells in the bottom side of the Transwell (Figure 3A). VEGFR1-Fc was added to controls to remove any trace amounts of VEGF-B produced by tumor cells. Endothelial cells were carefully removed prior to imaging of BODIPY-C16 fluorescence within tumor cells. “No TCs” served as negative control, demonstrated by the lack of fluorescence detected in the tumor cell–free panel, whereas “No ECs” served as positive control that allows tumor cells to take up the maximal amount of BODIPY-C16 (Figure 3B). The presence of WT endothelial cells significantly decreased BODIPY-C16 uptake by tumor cells relative to controls lacking endothelial cells (“No ECs,” Figure 3B), indicating that endothelial cells act as a physical barrier to BODIPY-C16. When WT endothelial cells are present, VEGF-B resulted in enhanced BODIPY-C16 uptake by tumor cells compared with the VEGFR1-Fc control (Figure 3, B and C). In contrast, tumor cell BODIPY-C16 uptake was unaffected by VEGF-B with *Rptor*-KO endothelial cells (Figure 3, B and C), indicating the requirement of mTORC1 activity to support transendothelial fatty acid delivery. Endothelial permeability was not significantly impacted by VEGF-B or *Rptor* loss (Supplemental Figure 4, D and E), suggesting that LCFAs are not delivered via diffusion. In agreement with a transcytotic mechanism, confocal microscopy revealed a lower percentage of cellular BODIPY-C16 signal at the basolateral region of *Rptor*-KO endothelial cells (Figure 3D). Upon hyperactivation of mTORC1 via *Tsc2* deletion, increased transendothelial delivery of BODIPY-C16 to tumor cells was observed even in the presence of VEGFR1-Fc, while VEGF-B had no further impact (Supplemental Figure 4F), together indicating that mTORC1 enhances transendothelial LCFA delivery through a VEGF-B–dependent transcytotic mechanism.

To evaluate whether endothelial mTORC1 promotes LCFA delivery to tumors in vivo, we examined BODIPY-C16 uptake in lung metastatic tumors derived from WT or *Rptor*^ECKO^ mice. BODIPY-C16 fluorescence intensity was significantly reduced in endothelial cells from *Rptor*^ECKO^ mice, while *Tsc2*^ECKO^ tumor endothelial cells exhibited elevated uptake (Figure 3E and Supplemental Figure 4, G and H). Likewise, BODIPY-C16 uptake in the tumor cell–enriched population was lower in *Rptor*^ECKO^ tumors but higher in *Tsc2*^ECKO^ tumors (Figure 3F and Supplemental Figure 4I). In contrast, BODIPY-C16 uptake by tumor or endothelial cells was unaffected by endothelial cell–specific Rictor loss (Supplemental Figure 4, J and K), suggesting that endothelial mTORC1, but not mTORC2, regulates LCFA delivery into lung metastatic tumors.

### RAB- and CLSTN1-dependent endosomal trafficking of LCFAs is defective in endothelial cells upon loss of mTORC1.

**Figure 4:**
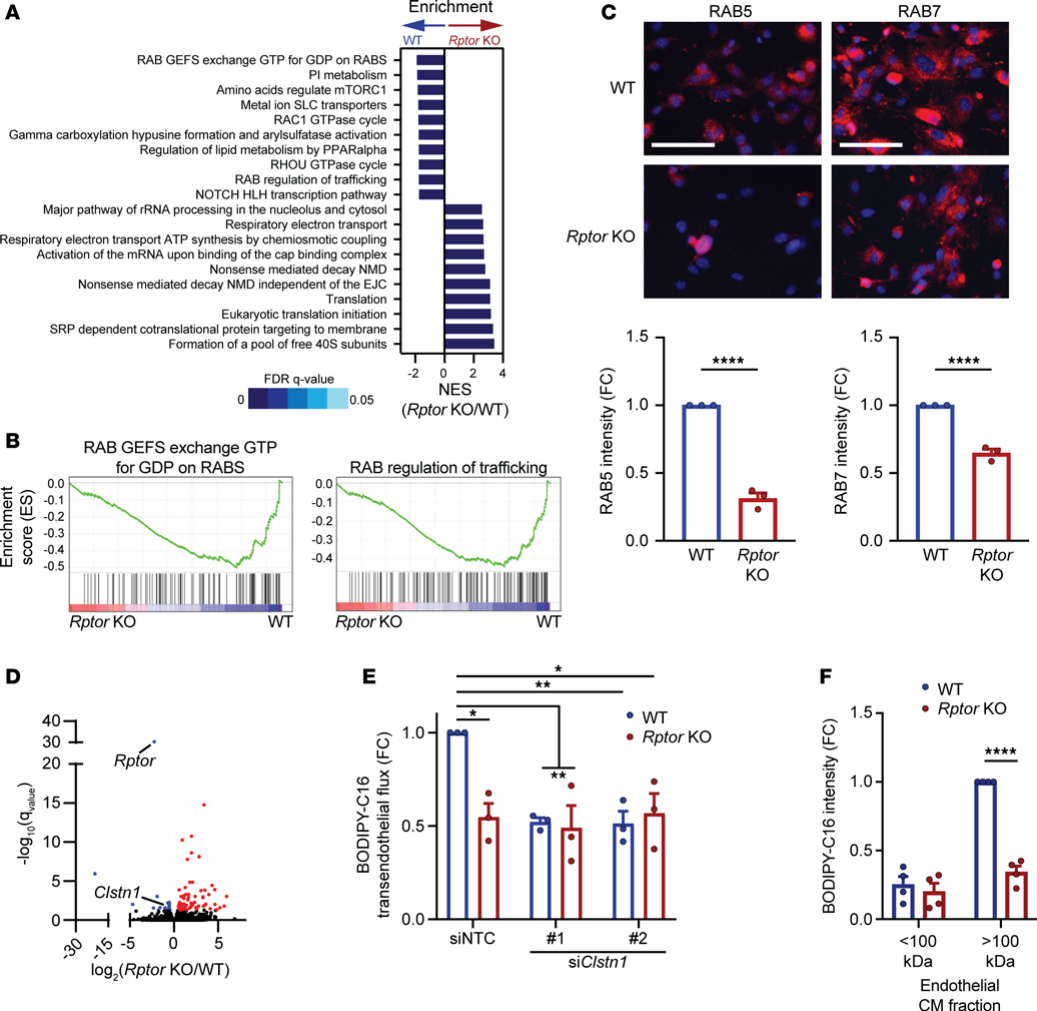
Raptor/mTORC1 loss reduces vesicle trafficking of fatty acids in endothelial cells. (**A** and **B**) Gene set enrichment analysis of WT or *Rptor*-KO endothelial cells (*n* = 4 per group). (**A**) Top enriched pathways are shown. Normalized enrichment score (NES) and false discovery rate (FDR) *q* values are indicated. (**B**) Enrichment plots for RAB trafficking pathways are shown. (**C**) Immunofluorescence of RAB5 (left) or RAB7 (right) (both red) was performed on WT or *Rptor*-KO endothelial cells (*n* = 3 per group). Representative images are shown. Nuclei were stained with DAPI (blue). Scale bars: 100 μm. (**D**) Gene expression volcano plot from data in **A** and **B**. Differentially upregulated genes in *Rptor*-KO cells are displayed in red, while downregulated genes are in blue. (**E**) Transendothelial transport of BODIPY-C16 in WT or *Rptor*-KO endothelial cells transfected with siRNA against *Clstn1* (*n* = 3 per group). BODIPY-C16 intensity in LLC tumor cells was normalized to the non-targeting control (NTC) and presented as fold change (FC). (**F**) LLC tumor cells were cultured in fractionated conditioned media from WT or *Rptor*-KO endothelial cells (*n* = 4 per group). *P < 0.05, **P < 0.01, ****P < 0.001 by unpaired, 2-tailed Student’s *t* test (**C**), 2-way ANOVA with Tukey’s post hoc test (**E**), or 2-way ANOVA with Šidák’s multiple-comparison post hoc test (**F**).

RNA-seq analysis of *Rptor*-KO lung microvascular endothelial cells revealed a downregulation of genes associated with RAB endosomal trafficking (Figure 4, A and B). Immunofluorescence imaging demonstrated a significant reduction in RAB5, a marker of early endosomes, and RAB7, a marker of endosome maturation, in *Rptor*-KO endothelial cells (Figure 4C). Alternatively, RAB5 and RAB7 were significantly increased in *Tsc2*-KO endothelial cells (Supplemental Figure 5A), indicating the importance of mTORC1 in endosomal trafficking in endothelial cells. Interestingly, VEGF-B exacerbated the deficiencies of RAB5 and RAB7 endosomes in *Rptor*-KO endothelial cells (Supplemental Figure 5B). Further transcriptome analysis of *Rptor-*KO endothelial cells revealed a downregulation of a key mediator of anterograde vesicle transport, calsyntenin-1 (*Clstn1*) (Figure 4D and Supplemental Figure 5C) (22). The CLSTN1 protein level was modestly reduced in *Rptor*-KO endothelial cells, while the presence of VEGF-B exacerbated the difference in CLSTN1 protein levels (Supplemental Figure 5D). In contrast, *Tsc2*-KO endothelial cells exhibited elevated *Clstn1* expression (Supplemental Figure 5E). Together, these data suggest that *Rptor*-KO endothelial cells exhibit defective vesicular trafficking.

We next examined whether changes in endosomal trafficking are responsible for the defects in transendothelial LCFA delivery in *Rptor*-KO endothelial cells. Knockdown of *Clstn1* in WT endothelial cells significantly reduced transendothelial delivery of BODIPY-C16 (Figure 4E and Supplemental Figure 5, F and G). While BODIPY-C16 transport was significantly reduced in *Rptor*-KO endothelial cells, *Clstn1* knockdown did not further alter BODIPY-C16 intensity in tumor cells (Figure 4E and Supplemental Figure 5G), suggesting that CLSTN1 functions downstream of mTORC1 to support transendothelial LCFA transport. While lipid delivery between cells has been reported to occur via free fatty acids, recent reports have indicated that fatty acids can be packaged into lipid-containing vesicles (23, 24). To evaluate whether LCFAs are exported as vesicle cargo, we collected conditioned medium from WT or *Rptor*-KO endothelial cells treated with BODIPY-C16 and further fractionated it based on size. Most soluble proteins and free fatty acids are present in the <100K fraction, while larger proteins and vesicles are sequestered to the >100K fraction (23, 24). LLC tumor cells were then incubated with fractionated conditioned endothelial cell media and BODIPY-C16 fluorescence was determined. The majority of BODIPY-C16 was located within the >100K fraction from WT endothelial cells, but the intensity was significantly reduced in tumor cells cultured with the >100K fraction from *Rptor*-KO endothelial cells (Figure 4F and Supplemental Figure 5H). Very little fluorescence was observed in the <100K fraction, suggesting that most BODIPY-C16 is packaged and exported in lipid-filled vesicles. Together, these data support a role for mTORC1 in driving transendothelial delivery of LCFAs via a vesicle-mediated mechanism.

To understand whether mTORC1 may regulate RAB/CLSNT1-dependent endothelial trafficking in human tumors, we examined the transcriptome profiles of human tumor–associated endothelial cells in breast cancer, lung cancer, and melanoma (Supplemental Figure 6). Expression of genes associated with mTORC1 signaling positively correlated with those involved in RAB regulation of trafficking (Supplemental Figure 6, A–C). Although CLSTN1 is most closely associated with vesicle trafficking in neurons (22), we observed expression in the endothelial cells within tumors, as well as tumor cells and fibroblasts (Supplemental Figure 6, D–G). Expression of *CLSTN1* in endothelial cells was positively associated with a higher mTORC1 expressional profile, which was stronger in tumor-associated endothelial cells (Supplemental Figure 6, H–J). Greater *CLSTN1* relative to the vascular marker *PECAM1* (CD31) was associated with poorer survival in patients with metastatic breast cancer (Supplemental Figure 6K), suggesting that endothelial *CLSTN1* may be a negative prognostic marker for metastatic breast cancer.

### Trafficking of fatty acids into the tumor supports tumor cell proliferation and invasion.

Given the role of endothelial mTORC1 in transporting fatty acids, we next evaluated whether lipids differentially accumulate in control and *Rptor*^ECKO^ tumors. Indeed, fewer neutral lipids were stored in *Rptor*^ECKO^ lung metastatic tumors (Supplemental Figure 7A), further indicating that endothelial mTORC1 regulates LCFA content in lung metastatic tumors. *Rptor*^ECKO^ tumors also exhibited a reduction in phosphorylated histone H3 (p-H3), a marker of mitosis (Supplemental Figure 7B). To evaluate whether accumulating fatty acids may enhance tumor growth and progression, we assessed how the LCFA palmitate impacts proliferation and invasion in vitro. Indeed, the presence of palmitate increased p-H3 and enhanced matrix invasion of embedded spheroids (Supplemental Figure 7, C and D). Therefore, by trafficking fatty acids into the metastatic microenvironment, the endothelium may have a direct impact on tumor outgrowth.

In response to the lower fatty acid composition, tumor cells within *Rptor*^ECKO^ tumors were also found to exhibit an altered metabolic profile. RNA-seq on sorted GFP^+^ tumor cells from WT or *Rptor*^ECKO^ metastatic tumors revealed that *Rptor*^ECKO^ tumor cells had lower expression of genes associated with the respiratory electron transport chain (ETC) (Supplemental Figure 7, E–G). Indeed, *Rptor*^ECKO^ lung metastatic tumors exhibit reduced levels of the ETC complex I subunit, NADH dehydrogenase (ubiquinone) Fe-S protein 1 (NDUFS1) (Supplemental Figure 7H). Given that fatty acid oxidation is closely associated with the ETC through production of NADH and FADH_2_ that are necessary for oxidative phosphorylation (25, 26), these findings suggest that reduced fatty acid delivery in *Rptor*^ECKO^ tumors induces a metabolic shift away from fatty acid catabolism that may directly reduce metastatic outgrowth.

### Loss of endothelial mTORC1 reduces fatty acid uptake and improves cytotoxic activation of T lymphocytes in metastatic tumors.

**Figure 5:**
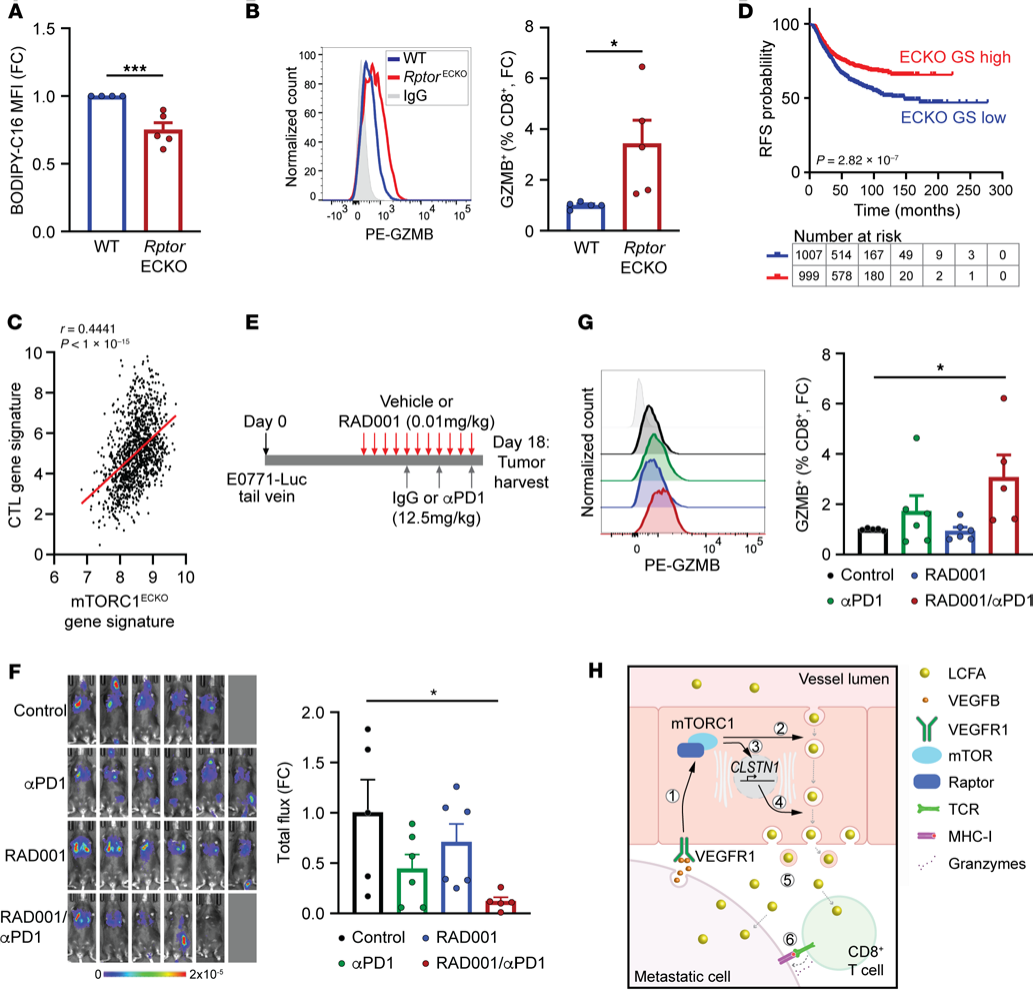
Targeting endothelial mTORC1 reduces fatty acid uptake and improves antitumor immunity to reduce lung metastatic outgrowth. (**A**) BODIPY-C16 MFI of CD8^+^ T cells from LLC metastatic tumors in WT (*n* = 4) or *Rptor*^ECKO^ (*n* = 5) male mice. (**B**) GZMB^+^CD8^+^ T cells in E0771-luc lung metastatic tumors from WT (*n* = 5) or *Rptor*^ECKO^ (*n* = 5) female mice. (**C**) Correlation of the mTORC1^ECKO^ and cytotoxic T lymphocyte (CTL) gene signatures in TCGA-BRCA. (**D**) Breast cancer recurrence-free survival (RFS) in the TCGA BRCA (*n* = 1218) dataset, stratified by mTORC1^ECKO^ gene signature (ECKO GS). Number of at-risk patients in each group is shown. HR is 0.6679 (95% CI = 0.5732–0.7782). (**E**–**G**) Pharmacological mTORC1 inhibition combined with αPD-1 immunotherapy. Control (*n* = 5), αPD-1 (*n* = 6), RAD001 (*n* = 6), RAD001/αPD-1 (*n* = 5). (**E**) Schematic of experimental procedures. (**F**) Bioluminescence of lung tumors on day 17. (**G**) GZMB^+^CD8^+^ T cells were determined by flow cytometry and normalized to Control. (**H**) Proposed model of long-chain fatty acid (LCFA) transport across endothelial cells into tumor tissue. (a) VEGF-B activates mTORC1, leading to (b) trafficking of LCFAs via RABs. (c) *CLSTN1* expression (d) promotes anterograde transport of LCFA-filled cargos. (e) Released LCFAs are taken up by cancer cells and CD8^+^ T cells, (f) suppressing cytotoxicity. *P < 0.05, ***P < 0.005 by unpaired, 2-tailed Student’s *t* test (**A** and **B**) or 1-way ANOVA with Dunnett’s post hoc test (**E**–**G**).

In addition to the direct impacts on tumor cells, accumulation of fatty acids has been associated with loss of antitumor functionality in CD8^+^ T lymphocytes (5, 6). Therefore, we next examined whether targeting endothelial mTORC1 may reduce fatty acid uptake by T cells in metastatic tumors. WT or *Rptor*^ECKO^ mice bearing lung metastatic tumors were injected with BODIPY-C16 prior to harvest. Flow cytometric analysis of harvested tumors revealed a significant reduction in BODIPY-C16 intensity within CD8^+^ tumor-infiltrating lymphocytes (TILs), without dramatically changing the percentage of BODIPY^+^CD8^+^ T cells (Figure 5A and Supplemental Figure 8A). Similarly, CD4^+^ T cells exhibited a significant decrease in BODIPY-C16 intensity, but also fewer positive cells (Supplemental Figure 8, B and C). In contrast, both CD8^+^ and CD4^+^ T cells from *Tsc2*^ECKO^ mice contained greater BODIPY-C16 fluorescence than controls (Supplemental Figure 8, D and E), demonstrating that endothelial mTORC1 can regulate T cell LCFA uptake.

We next evaluated how endothelial mTORC1 impacts T cell numbers and function within metastatic tumors. Although the overall T cell composition of *Rptor*^ECKO^ and *Tsc2*^ECKO^ tumors was not substantially altered (Supplemental Figure 8, F–M), we did observe changes in T cell function. More CD8^+^ T cells in *Rptor*^ECKO^ tumors expressed markers of cytolytic activity, including granzyme B (GZMB), CD107a, and the cytokines TNF-α and IFN-γ (Figure 5B and Supplemental Figure 9, A–D). Similarly, fewer CD8^+^ T cells exhibiting markers of exhaustion were observed in *Rptor*^ECKO^ lung metastatic tumors (Supplemental Figure 9, E and F), consistent with greater functionality upon loss of endothelial mTORC1. No change was observed in cytolytic markers of CD8^+^ T cells from *Tsc2*^ECKO^ tumors (Supplemental Figure 9, G and H). Consistent with decreases in fatty acid content improving antitumor T cell responses, the cytotoxic capacity of CD8^+^ T cells decreases in the presence of palmitate (Supplemental Figure 9, I and J). In contrast with CD8^+^ TILs, elevated fatty acid uptake improves survival and function of T regulatory cells (Tregs) (27). Indeed, activated Tregs characterized by high CD25 and low CD127 expression were significantly decreased in *Rptor*^ECKO^ tumors, but not in *Tsc2*^ECKO^ tumors (Supplemental Figure 9, K and L). Together, these findings suggest that endothelial mTORC1 promotes fatty acid delivery to create an immunosuppressive microenvironment during metastatic outgrowth.

To assess the relationship between endothelial mTORC1 activity and CTL responses and survival from publicly available bulk patient samples, we used transcriptome changes observed in *Rptor*^ECKO^ tumor cells (Supplemental Figure 7G) to develop a gene signature. The mTORC1^ECKO^ gene signature reflects the expression profile of tumor cells that are associated with low endothelial mTORC1 activity. Indeed, a CTL gene signature (28) was positively correlated with the mTORC1^ECKO^ gene signature in breast cancer, lung cancer, and melanoma datasets (Figure 5C and Supplemental Figure 10, A and B). Similar findings were observed in metastatic cancer datasets from breast cancer and melanoma (Supplemental Figure 10, C and D). Patients with tumors exhibiting transcriptional profiles similar to mTORC1^ECKO^ tumors also incur markedly better recurrence-free survival (RFS) probabilities and improved responses to pembrolizumab (αPD-1) therapy (Figure 5D and Supplemental Figure 10, E and F). These data reflect enhanced antitumor immune responses and survival in patients with low endothelial mTORC1 activity.

### Low-dose RAD001 in combination with αPD-1 therapy reduces fatty acid delivery and metastatic tumor burden.

Although the mTORC1 inhibitor everolimus (RAD001) has been long considered to be immunosuppressive, the combination of RAD001 or other rapalogs with αPD-1 therapy has shown some benefit in murine tumor models, even at immunosuppressive doses (29–31). Recent studies have demonstrated that low doses can inhibit mTORC1 signaling without causing immunosuppression (21, 32, 33), and patient data suggest that targeting endothelial mTORC1 may improve αPD-1 responses (Supplemental Figure 10F). We previously demonstrated that use of low-dose RAD001 selectively inhibited mTORC1 in endothelial cells, without affecting mTORC1 signaling in immune cells or tumor cells (21). Therefore, we evaluated whether low-dose RAD001 in combination with αPD-1 therapy would decrease LCFA delivery and reduce lung metastatic outgrowth (Figure 5E). Compared with vehicle/IgG control, lung tumor burden was significantly reduced in animals treated with the RAD001/αPD-1 combination therapy (Figure 5F and Supplemental Figure 11, A–C). Indeed, RAD001 alone or in combination with αPD-1 reduced p-S6 levels in endothelial cells but did not dramatically reduce Ki67 positivity (Supplemental Figure 11, D and E), suggesting that endothelial cell proliferation is not affected under these treatment conditions. RAD001/αPD-1 significantly reduced BODIPY-C16 fluorescence in both endothelial and tumor-enriched cell populations, while a similar decrease was observed in endothelial cells of RAD001-treated mice (Supplemental Figure 11, F and G), consistent with less LCFA transport into metastatic tumors upon mTORC1 inhibition. Likewise, the combination of RAD001 with αPD-1, but not either agent alone, significantly reduced BODIPY-C16 uptake by CD4^+^ and CD8^+^ T cells, while increasing T cell enrichment and cytolytic GZMB^+^ CTLs (Figure 5G and Supplemental Figure 11, H–L). Together, these results suggest that endothelial mTORC1 can be therapeutically targeted to improve antitumor responses in combination with αPD-1 therapy to treat metastatic progression (Figure 5H).

## Discussion

The changing nutrient composition within the early metastatic microenvironment is a critical factor in metastatic progression. Recent studies have demonstrated the importance of fatty acids in metastasis, including their enrichment in the lung metastatic microenvironment (2–4). However, the mechanism by which fatty acids are delivered to early metastatic tumors is poorly understood. The endothelium serves as a gatekeeper between the nutrient-rich blood and highly metabolic tissues. Here, we report that endothelial mTORC1 is a key driver of transendothelial fatty acid delivery to early metastatic tumors, leading to reduced antitumor activities by T cells to support metastatic outgrowth (Figure 5H). Selective pharmacological inhibition of endothelial mTORC1 using a low dose of everolimus/RAD001 enhanced the efficacy of PD-1 inhibition that reduced metastatic outgrowth, with a concomitant decrease in LCFA enrichment and elevated cytolytic capacity in T cells. These findings improve our understanding of how fatty acid composition is regulated in the early metastatic microenvironment and demonstrate that selective endothelial mTORC1 inhibition may improve immune checkpoint therapy responses in metastatic disease.

LCFAs have been found to accumulate during tumor progression, including at the metastatic site (3, 5). Multiple sources of fatty acids within tumors have been described, including stromal cell release and de novo synthesis within tumor cells (3, 34). However, several groups have linked elevated dietary intake of lipids with cancer progression (3, 35, 36), suggesting that the blood may be a substantial source of free fatty acids in tumors. Endothelial cells that line blood vessels serve as gatekeepers to regulate dispersal of nutrients and other molecules into surrounding tissues via transendothelial delivery (7, 8). Indeed, our data demonstrated that endothelial cells are responsible for delivery of LCFAs into the TME through an mTORC1-dependent mechanism. Using a murine model of metastatic outgrowth, loss of the mTORC1 component Raptor in endothelial cells reduced uptake of the fluorescent palmitate analog BODIPY-C16 within tumors that was associated with a decrease in metastatic tumor burden. These findings support fatty acid delivery from the blood as a substantial source during metastatic progression within the lung. It remains unclear whether elevated dietary fats may increase fatty acids inside metastatic tumors through a mTORC1-dependent endothelial delivery mechanism. However, the highly vascular nature of lung suggests that transendothelial delivery may provide a substantial source of fatty acids for disseminated tumor cells within the lung metastatic niche. While a similar mechanism may also contribute to other metastatic sites, such as bone and brain, the use of a tail-vein injection model limits evaluation at these important secondary sites. Furthermore, it is unclear whether specialized fenestrated sinusoidal endothelial cells within the liver, which serve to readily diffuse substrates from the blood, would undergo vesicular transport of fatty acids.

While tumor angiogenesis through VEGF-A and VEGFR2 has been extensively studied, the role of VEGF-B and its cognate receptor VEGFR1 is less understood. In normal but highly metabolic tissues, VEGF-B has been described as a driver of transendothelial fatty acid delivery (7, 8), although the signaling mechanism has not been defined. Our findings describe a critical role of mTORC1 in VEGF-B–associated transport of LCFAs across endothelial cells in lung metastasis. While VEGFR1 is necessary for VEGF-B activation of mTORC1, neuropilin-1 (NRP1) is also required for fatty acid transport across endothelial cells, but it is unclear how this co-receptor may be involved in the signaling cascade (7, 37–40). Like skeletal muscle, heart, and adipose tissues, VEGF-B expression is elevated in human disease, including increased expression within human and murine cancer cell lines, such as LLC cells used in this study (9–11, 41). Still, its role in cancer has been largely unknown, which may be somewhat attributed to both pro- and antiangiogenic properties described for VEGF-B (12–14). It remains possible the VEGF-B isoforms, including the soluble VEGF-B_186_ and membrane-bound VEGF-B_167_, may have different properties. Most studies have focused on the shorter isoform, which is more broadly expressed (9). However, we evaluated VEGF-B_186_, which is more highly expressed in tumors and cancer cell lines and was found to have a more profound impact on fatty acid uptake in endothelial cells (7), supporting the notion of a differential isoform effect. Although VEGF-B does not appear to dramatically impact primary tumor growth, a study that evaluated the full-length gene found that VEGF-B does support the development of metastasis in the lungs (11). Our data are consistent with a role of VEGF-B in promoting early metastatic outgrowth by creating a fatty acid–rich environment within the early lung metastatic niche. Interestingly, Yang and colleagues attributed the VEGF-B–associated metastatic spread to a decline in vessel integrity (11). We previously demonstrated that Raptor loss in endothelial cells improves tumor vessel structure and reduces metastatic dissemination but has no considerable impact on the lung vascular bed in non–tumor-bearing animals (21). Although additional studies will be necessary to elucidate the mechanism of mTORC1 in vessel integrity, it is intriguing to speculate that changes in VEGF-B signaling or fatty acid content may contribute to mTORC1’s role in vessel function.

Endocytic trafficking across the endothelial cell lining supports delivery of nutrients to surrounding tissues, although the mechanisms regulating this process have not been thoroughly explored. Most studies have investigated those impacting the blood-brain barrier, where PTEN, a negative regulator of mTORC1 signaling, was identified as a suppressor of endothelial transcytosis (42). Our data demonstrate that mTORC1 promotes fatty acid uptake and endosomal transcytosis in microvascular lung endothelial cells to mediate transendothelial fatty acid delivery during metastatic outgrowth. Indeed, endothelial transcytosis involves RAB7 late endosomes, feeding into multivesicular bodies that release small extracellular vesicles, both of which contain palmitate as cargo (43–45). We also demonstrated that mTORC1 regulated expression of CLSTN1, a protein implicated in endosomal trafficking and exocytosis (22). Early studies attributed the mechanism to increased fatty acid uptake, given that VEGF-B promoted expression of the fatty acid transporters FATP3 and FATP4 (7). Recent studies have suggested that FATP3 and FATP4 may serve to sequester fatty acids, particularly along the mitochondria and endoplasmic reticulum, respectively, where these proteins are localized (46–49). This localization has sizable implications for endothelial metabolism, especially given the acyl-CoA synthetase activity of the FATP family (49, 50). We observed intense perinuclear BODIPY-C16 localization in endothelial cells, although this compartmentalization did not appear to be dramatically impacted by mTORC1 status. It remains possible that autophagy activation in the absence of mTORC1 may maintain fatty acid uptake (51), but the overall reduction in LCFAs in Raptor-deficient endothelial cells may have secondary impacts on endothelial cell fatty acid metabolism. However, metabolomics analysis revealed no substantial changes in complex lipids or fatty acid metabolism (Supplemental Table 1), suggesting that mTORC1 may not dramatically alter fatty acid metabolism in endothelial cells. Still, in addition to its role in lipid transport, VEGF-B and mTORC1 have been shown to have secondary effects on general endothelial sugar and amino acid metabolism (7, 52, 53) that may have major impacts on vessel structure and function in tumors (54–56). Additional studies are necessary to more completely understand whether endothelial metabolism may contribute to endothelial function in response to targeting mTORC1.

In addition to providing fatty acids to serve as fuel for proliferating tumor cells, a growing body of evidence suggests that accumulating fatty acids within tumors has substantial negative impacts on antitumor immunity. Although CD8^+^ TILs do utilize LCFAs to support effector function in some environments, those highly enriched with fatty acids lead to dysfunction (5). Within environments enriched for LCFAs, Manzo and colleagues demonstrated that CD8^+^ T cells underwent transcriptional changes that impair their ability to utilize and safely store fatty acids (5). The presence of palmitate reduced the ability of CD8^+^ T cells to produce effector cytokines and cytolytic enzymes, including IFN-γ, TNF-α, and GZMB (5). Excessive LCFA uptake also promotes lipid peroxidation and ferroptosis in CD8^+^ T cells and drives immunosuppressive CCL2 production within the TME that may have additional protumor influences on the vasculature (6, 57–59). Further potentiating an immunosuppressive microenvironment, high fatty acid levels are supportive of Treg cell survival (27). In agreement, our findings demonstrated that loss of Raptor/mTORC1 in endothelial cells reduced fatty acid uptake by TILs, improved antitumor CD8^+^ T cell effector function, and reduced immunosuppressive Tregs during metastatic outgrowth. Interestingly, these improvements in antitumor lymphocyte responses may further enhance tumor cell death mediated by ferroptosis (60). Additional immunosuppressive influences of accumulating fatty acids within the diverse TME have been described in myeloid populations that may have further implications for T cell responses. Within palmitate-enriched conditions, dendritic cells exhibit impaired antigen presentation, while tumor-associated macrophages undergo a metabolic switch toward fatty acid oxidation to support M2 polarization (61–65). Thus, reducing fatty acid accumulation within the TME may improve antitumor immune responses.

Metastatic cancer remains uncurable, but recent advances in immunotherapy have offered improved outcomes in some patients. Unfortunately, the overall response rate remains low, particularly those with tumors considered immunologically cold (66). In recent years, vasculature-targeting strategies, such as antiangiogenics, have been shown to improve immunotherapy responses in both preclinical models and patients with advanced cancer (67–74). As a major contributor to immunosuppression, the metabolic TME has also been an area of intense research to identify new targets to improve immunotherapy responses (75–78). Our findings suggest that fatty acid accumulation within metastatic tumor lesions can be reversed by targeting endothelial mTORC1 to improve immunotherapy efficacy. We demonstrate that selective pharmacological inhibition of endothelial mTORC1 using low doses of everolimus/RAD001 improved αPD-1 responses that correlated with reduced LCFA uptake by T lymphocytes. Compared with either drug alone, the combination of low-dose RAD001 with αPD-1 showed the strongest effects on T cells, reducing fatty acid uptake and improving cytolytic capacity. PD-1 signaling is also associated with a metabolic switch in CD8^+^ T cells toward fatty acid uptake and metabolism, leading to mitochondrial damage and ferroptosis in lipid-enriched environments (79, 80). While a reduction in T cell BODIPY-C16 uptake appears to be partially attributable to pharmacological targeting of endothelial mTORC1, our data suggest that αPD-1 therapy may further improve the metabolic fitness of TILs that supports further improvements in antitumor immunity in lung metastatic tumors. Although everolimus/RAD001 dose reduction strategies have been largely ineffective in cancer patients while still being associated with some severe toxicities, it is important to note that our study used a dose that is approximately 10-fold lower than low doses used in patients (81–83). Therefore, our findings suggest that vasculature-targeting strategies to reduce environmental fatty acid accumulation may represent a new approach to enhancing immunotherapy responses in metastatic cancer.

## Methods

Further information can be found in Supplemental Methods.

### Sex as a biological variable.

Breast cancer primarily affects women. Therefore, breast cancer models used only female mice. Male mice were used with LLC cells.

### Cell culture.

LLC and E0771 parental cells were provided by Barbara Fingleton (Vanderbilt University). Mouse mammary tumor virus–driven polyoma middle T antigen (MMTV-PyMT) cells were isolated from primary murine C57BL/6 mammary tumors (84) and provided by Rebecca Cook (Vanderbilt University). LLC, E0771, and MMTV-PyMT parental cells were maintained as previously described (21, 28, 54). GFP and luciferase were cloned into the pCDH-puro plasmid. The lentiviral vector pLX311-luciferase was a gift from William Hahn (Addgene, plasmid 117735). Parental cells were transduced with pCDH-GFP-luc or pLX311-luc. GFP^+^ cells were enriched using cell sorting. E0771-OVA cells were generated by overexpression of ovalbumin (OVA) as previously described (28). Murine pulmonary microvascular endothelial cells (MPMECs) were isolated from 8- to 16-week-old *Rptor^fl/fl^* or *Tsc2^fl/fl^* mice and maintained in EGM-2 medium (Lonza), as previously described (85–89). For adenoviral Cre expression, MPMECs were seeded onto tissue culture plates coated with 0.1% gelatin at 70%–80% confluence. Cells were infected with 1 × 10^7^ PFU/mL of Ad-CMV-iCre (Vector Biolabs, 1045) for 16–48 hours, as indicated. Ad-CMV-b-Gal (Vector Biolabs, 1080) and Ad-CMV-Null (Vector Biolabs, 1300) were used as control vectors as indicated.

### Animals.

All mice used in this study were maintained on a C57BL/6 background and housed in a non-barrier facility. WT female C57BL/6 and OT-I mice were purchased from The Jackson Laboratory. CDH5-CreER^T2^ mice and animals with the *Rptor^fl/fl^* or *Rictor^fl/fl^* allele were sourced and maintained as described previously (21, 85). *Tsc2^fl/fl^* mice were generated and provided in-house (90). Animals were genotyped for Cre or floxed alleles of *Rptor*, *Rictor*, or *Tsc2* alleles using the primers listed in Supplemental Table 2. To induce endothelial cell–specific deletion of *Rptor*, *Rictor*, or *Tsc2*, tamoxifen (Sigma-Aldrich, T5648) was reconstituted in sunflower seed oil (Sigma-Aldrich, S5007) and administered intraperitoneally (i.p.) for 5 consecutive days in 7- to 12-week-old mice at a final dose of 2 mg/mouse, as previously described (21). Tamoxifen was administered beginning 4 days after intravenous tumor cell inoculation. For studies in tumor-free animals, lungs were harvested 7 days after the final tamoxifen dose.

### Tumor models.

For modeling metastatic outgrowth, 1 × 10^6^ cancer cells in 200 μL PBS were intravenously injected into the tail vein. For VEGFR1 neutralization in vivo, animals were treated with 2.5 mg/kg of normal rabbit IgG (R&D Systems, AB-108-C) or αVEGFR1 (R&D Systems, AF471) every 3–4 days by i.p. injection. Lung metastatic tumors were tracked weekly in live animals with bioluminescence imaging (IVIS Spectrum, PerkinElmer). Animals were sacrificed on day 14–18, and lungs were perfused with PBS through the cardiac left ventricle. Lung weights were recorded, and gross lungs imaged using an Olympus stereo microscope. GFP intensity was measured using ImageJ software (NIH) as arbitrary units (AU), using the mean intensity from front and back side of each lung. Total radiance flux (photons per second within a cm^2^ tissue area per steradian, p/sec/cm^2^/st) was calculated in the thorax and normalized to WT littermate controls using Living Image v.4.8.2 (PerkinElmer) or Aura v4.0.8 (Spectral Images) software. To assess fatty acid uptake of tumor cell populations, animals were inoculated and induced with parental or luciferase-expressing tumor cells as described above. Beginning 1 hour prior to sacrifice and harvest, animals were injected with 50 μg of BODIPY-C16 (BODIPY FL C16, Thermo Fisher Scientific, D3821) in 50 μL DMSO by i.p. injection.

For drug studies with everolimus/RAD001 and αPD-1, E0771-luc cells were injected into 8-week-old WT C57BL/6 female mice, as described above. Seven days after tumor inoculation, mice were treated with vehicle+IgG, vehicle+αPD-1, RAD001+IgG, or RAD001+αPD-1. Vehicle (20% DMSO in PBS) or 0.01 mg/kg RAD001 (Selleck Chemical, S1120) was administered daily in 100 μL. IgG (250 μg; BioXCell, BE0089) or αPD1 (250 μg; clone RMP1-14, BioXCell, BE0146) was administered i.p. in 100 μL PBS every 3 days beginning on day 11. Bioluminescent imaging and BODIPY-C16 treatment were performed as above.

### Metabolite profiling.

MPMECs isolated from *Rptor^fl/fl^* mice were transduced with LacZ- or Cre-expressing adenovirus for 48 hours, and cells were collected 24 hours later. Cell pellets were profiled by Metabolon using ultra-high-performance LC-MS/MS to detect 541 metabolites. Compounds were identified against a library of standards, and abundance was normalized to protein content. Scaled results were grouped in major classes and subclasses. Four samples from independent MPMEC isolations were analyzed in each group.

### Flow cytometry.

Lung metastatic tumors were dissociated in RPMI-1640, 5% FBS, collagenase IA (1 mg/mL; Sigma-Aldrich, C9891), and DNase I (0.25 mg/mL; Sigma-Aldrich, DN25), filtered through a 70 μm cell strainer, and red blood cells were lysed, as previously described (28). For detection of IFN-γ and TNF-α, 2 × 10^6^ live cells were stimulated in RPMI-1640 supplemented with 5% FBS, phorbol 12-myristate 13-acetate (PMA) (50 ng/mL; Sigma-Aldrich, P8139), ionomycin (1 μg/mL; Sigma-Aldrich, I0634), and GolgiStop protein transport inhibitor (1:1500; BD Biosciences, 554724) at 37°C for 4 hours. To exclude dead cells from analysis, Ghost Dye Violet 510 (Tonbo Biosciences, 13-0870) was used. Blocking with αCD16/αCD32 (Tonbo, 70-0161) was followed by staining of the following extracellular proteins with antibodies shown in Supplemental Table 3. A Cytofix/Cytoperm solution kit (BD Biosciences, 554714) was used to the detect intracellular GZMB, IFN-γ, TNF-α, and FABP3 per manufacturer’s instructions. The FoxP3/Transcription Factors Staining Kit (Tonbo, TNB-0607) was used to detect FoxP3, as directed. To detect unconjugated antibodies, a subsequent incubation with secondary Alexa Fluor 647 goat anti-rabbit (1:200; Invitrogen, A21244) was performed. BODIPY-C16 was detected using the Alexa Fluor 488 channel. To detect p-S6 and Ki67, 4 × 10^6^ cells were fixed in methanol and incubated with αp-S6, followed by incubation with additional markers as well as secondary Alexa Fluor 647 goat anti-rabbit as described previously (21). Tumor cell suspensions or isolated splenocytes were used for compensation, unstained, fluorescence minus one (FMO), and isotype controls, where appropriate. Using BD FACS Diva software, flow cytometry data were acquired on a BD Fortessa, and analysis was completed using FlowJo software (v10). Data collected from cultured cells was normalized to controls. In vivo data were normalized to littermate controls.

### Transendothelial delivery assay.

LLC parental cells (5 × 10^4^) were initially plated onto the bottom side of a 0.4 μm pore size Transwell insert (Corning, 3470). After LLC attachment, 1 × 10^5^ *Rptor^fl/fl^* or *Tsc2^fl/fl^* MPMECs were plated into the inner side coated with 0.1% gelatin. Cells were transduced with Ad-CMV-Null or Ad-CMV-iCre for 16 hours, followed by stimulation with VEGF-B_186_ (300 ng/mL) for 30 hours in EBM-2 supplemented with fatty acid–free 1% BSA (FAF-BSA). Control cells were incubated with soluble hVEGFR1 (VEGFR1-Fc, 1 μg/mL; R&D Systems, 321-FL-050) to bind tumor cell–derived VEGF-B. BODIPY-C16 (1 μM) was added to the top chamber for 1 hour, and cells were thoroughly washed 3 times with 1% FAF-BSA in EBM-2. An experimental control without endothelial cells (“No ECs”) was included to demonstrate that endothelial cells act as a barrier, limiting access of tumor cells to BODIPY-C16. An additional control without tumor cells (“No TCs”) was included to confirm adequate removal of endothelial cells prior to imaging. Cells were fixed with 4% paraformaldehyde (PFA) for 10 minutes and endothelial cells were carefully removed using a cotton-tip applicator. The Transwell lining with tumor cells was imaged using an Olympus compound microscope. BODIPY-C16 intensity was quantitated in ImageJ and normalized to WT+VEGFR1-Fc controls.

To evaluate transport after *Clstn1* knockdown, *Rptor^fl/fl^* MPMECs were transduced with empty or Cre-expressing adenovirus for 16 hours, followed by transfection with 40 nM ON-TARGETplus siClsnt1 (Horizon Dharmacon, J-044659-09 and J-044659-10) or non-targeting control (Horizon Dharmacon, D001810-10-20) using Lipofectamine RNAiMAX (Invitrogen, 13778150), per manufacturer’s instructions. After 24 hours, LLC cells and MPMECs were plated onto Transwells and assayed as described above.

### BODIPY confocal imaging.

*Rptor^fl/fl^* MPMECs were transduced with Ad-CMV-Null or Ad-CMV-iCre for 24 hours on MatTek dishes with a no. 1.5 coverslips coated with 0.1% gelatin. Cells were stimulated with VEGF-B (300 ng/mL) for 30 hours in EBM-2 supplemented with 1% FAF-BSA. Prior to imaging, live cells were stained with phalloidin–iFluor 594 (Abcam, ab176757) and Hoescht 33342 (Invitrogen, R37605), according to manufacturer’s instructions. Cells were incubated with BODIPY-C16 (20 μM) for 20 minutes at 4°C, and then washed 3 times in EBM-2 with FAF-BSA. Cells were then incubated at 37°C for 5 minutes and immediately imaged using a Nikon spinning disk microscope. Images from 3 regions were collected from 3 independent experiments. The *z*-plane representing the apical (top) and basolateral (bottom) edges of the cell were determined by phalloidin staining. The basolateral surface represents the bottom 10% of *z*-plane images for each cell. BODIPY-C16 intensity was quantitated in ImageJ, and the intensity present at the basolateral surface was calculated as a percentage of whole-cell intensity.

### Conditioned medium assay.

*Rptor^fl/fl^* MPMECs were transduced with Ad-CMV-Null or Ad-CMV-iCre for 16 hours, and then stimulated with VEGF-B (300 ng/mL) in EBM-2 supplemented with 1% FAF-BSA for 30 hours. Endothelial cells were incubated with 5 μM BODIPY FL C16 for 1 hour and subsequently thoroughly washed to remove free BODIPY. Cells were cultured in EBM-2 with 1% FAF-BSA for 16 hours. Conditioned medium was collected and fractionated using 100 kDa spin columns (Amicon, UFC510024), and resulting fractions were reconstituted to equivalent volumes. Complete or fractionated (<100 kDa or >100 kDa) conditioned medium was added to LLC tumor cells for 4 hours. Cells were washed thoroughly and images were captured from 3 fields of view using an Olympus microscope. BODIPY-C16 intensity was calculated using ImageJ and normalized to samples cultured in medium from the >100 kDa fraction from WT endothelial cells.

### Immunofluorescence.

*Rptor^fl/fl^* or *Tsc2^fl/fl^* MPMECs (2.5 × 10^4^ cells each) were seeded onto a 96-well plate coated with 0.1% gelatin and transduced with Ad-CMV-Null or Ad-CMV-iCre for 48 hours. For VEGF-B stimulation, *Rptor^fl/fl^* MPMECs were transduced as described above for 24 hours. Cells were then incubated with 1 μg/mL VEGFR1-Fc or 300 ng/mL VEGF-B for 30 hours in 1% FAF-BSA prior to immunofluorescence. Cells were fixed with 2% PFA and permeabilized with 1% Triton X-100. Cells were blocked with 3% BSA and probed with antibodies against TSC2 (Cell Signaling Technology, 4308), RAB5 (Cell Signaling Technology, 3547), or RAB7 (Cell Signaling Technology, 9367) using a 1:100 dilution overnight at 4°C. Cells were then incubated with Alexa Fluor 488 (Invitrogen, A11034) or Alexa Fluor 594 (Invitrogen, A11012) anti-rabbit secondary antibodies, both 1:500 for 1 hour. Nuclei were stained with DAPI (Invitrogen, R37606), as indicated. Three fields of view per sample were obtained using an Olympus inverted fluorescent microscope. Signal intensity was quantitated using ImageJ and normalized to the appropriate control.

To examine proliferation of tumor cells treated with palmitate, E0771 cells were incubated with 0 or 50 μM palmitate-BSA (Cayman Chemical, 29558) for 48 hours. Immunofluorescence was performed as described above, using anti–p-H3 (1:100; Cell Signaling Technology, 9701) and anti-rabbit Alexa Fluor 488 secondary antibody (1:500; Invitrogen, A11034). Nuclei were stained with DAPI. Five to 10 fields of view were obtained as described above and percentage of positive cells was determined using ImageJ and normalized to control.

### RNA-seq.

*Rptor^fl/fl^* MPMECs were transduced with Ad-CMV-Null or Ad-CMV-iCre for 48 hours. RNA was extracted using TRIzol (Life Technologies, 15596026) and an RNeasy Kit (Qiagen, 74104), as directed.

To isolate RNA from lung metastatic tumor cells, WT or *Rptor*^ECKO^ male mice were inoculated with LLC-GFP-luc cells. Dissociated tumor cells were separated based on CD45 using CD45 mouse microbeads (Miltenyi Biotec, 130-052-301), according to manufacturer’s instructions. GFP^+^ tumor cells were collected by flow sorting, and RNA was extracted using an RNAqueous Micro Total RNA Isolation Kit (Invitrogen, AM1931), according to manufacturer’s instructions. RNA-seq was performed by BGI Americas using the DNBSEQ platform. After sequencing, raw data were filtered to remove reads with high rates of unknown bases, low quality reads, and reads of adapter sequences. Clean reads were aligned to the reference genome (*Mus musculus*, version GCF_000001635.26_GRCm38.p6) using HISAT and aligned to reference genes using BowTie2. Differentially expressed genes (DEG) were identified using DESeq2 (*q* < 0.05) using the Dr. Tom platform (BGI Americas). Pathway enrichment analysis was performed against REACTOME gene sets using Gene Set Enrichment Analysis (GSEA) software (v4.3.2, Broad Institute) (91, 92).

### RNA human dataset analysis.

RNA expression data from TCGA Breast Adenocarcinoma (BRCA v.12-17-2021, *n* = 1218), TCGA lung cancer (LUNG v.05-26-2021, *n* = 1129), and TCGA Melanoma (SKCM v.11-02-2022, *n* = 474) were acquired from the UCSC Xena platform (https://xena.ucsc.edu) (93). RNA expression data of metastatic biopsies from The Metastatic Breast Cancer Project (www.mbcproject.org) (*n* = 18) and the Metastatic Melanoma (*n* = 38) (94) databases were downloaded from cBioPortal (https://www.cbioportal.org/) on February 8, 2023. Average log_2_-transformed expression of the mTORC1^ECKO^ and CTL gene sets (Supplemental Table 5) comprise each gene signature, respectively.

Single-cell RNA-seq data from breast cancer (NCBI Gene Expression Omnibus [GEO] GSE176078) (95), lung adenocarcinoma (96), and melanoma (GEO GSE72056) (97) patient datasets were used to analyze previously identified cell populations with Seurat v.4.9.9 (98). Gene set enrichment scores for endothelial cells were determined by performing ssGSEA on each single-cell expression matrix using GSVA in R (v.4.3.1) (99). REACTOME gene sets for “mTORC1-mediated signaling” and “RAB regulation of trafficking” (Supplemental Table 6) were identified from the curated C2 collection of the Molecular Signatures Database (MSigDB) (91, 100, 101). Individual endothelial cells were stratified based on the median mTORC1-mediated signaling enrichment score and *CLSTN1* expression was determined.

### Survival analysis.

RFS, first progression survival (FP), or progression-free survival (PFS) data in breast cancer (102), lung cancer (103), and melanoma (104), respectively, were downloaded from KM plot (https://www.kmplot.com/analysis/). Patients were stratified by mean expression of genes comprising the mTORC1^ECKO^ gene set in Supplemental Table 5, using the following expression cutoffs: breast cancer RFS (674.7, range = 265–2705), lung cancer FP (1132.65, range = 312–2733), no ICI melanoma PFS (1060.1, range = 488–5000), and pembrolizumab melanoma PFS (1165.49, range = 557–2805). Metastatic overall survival (OS) data was obtained from the AURORA metastatic breast cancer dataset (GEO GSE209998) (105). *CLSTN1* was normalized to vascular density using CD31/*PECAM1*. Patients were stratified by low (quartile 1–3) or high (quartile 4) *CLSTN1*/*PECAM1* ratio.

### Statistics.

All plots and statistical analyses were performed using GraphPad Prism software (v10.0.3). Individual data points are shown where possible. Summary data are mean ± SEM. For in vivo tumor experiments, data are reported from 2–4 independent experiments, where each point represents an individual animal. Statistical comparisons between 2 groups were performed using unpaired Student’s *t* test or Welch’s *t* test, as indicated. For multiple comparisons, 1- or 2-way analysis of variance (ANOVA) was performed with individual comparisons evaluated using Tukey’s, Dunnett’s, or Šidák’s post hoc analysis, as indicated. Outliers were excluded using the ROUT method (*Q* = 5%). All statistical analyses were 2-tailed, and differences with a *P* value of less than 0.05 were considered to be statistically significant.

For Kaplan-Meier survival curve analysis, log-rank analysis was performed between groups using the Mantel-Cox method. Hazard ratios (HR) and 95% confidence intervals (CI) were determined using the log-rank test. Pearson correlation (*r*) was performed on TCGA datasets.

### Study approval.

Studies involving animals were performed with approval from the Vanderbilt University Medical Center’s Institutional Animal Care and Utilization Committee (IACUC).

### Data availability.

Mouse RNA-seq data generated by this study have been deposited in the NCBI GEO and are available under the accession numbers GSE256508 (tumor cells) and GSE256509 (endothelial cells). Publicly available single-cell RNA-seq data used in this study can be found under the accession numbers GSE176078, GSE72056, and GSE209998, or at Code Ocean (doi:10.24433/CO.0121060.v1). Additional code used in this study for analysis of single-cell RNA-seq databases is available at https://doi.org/10.5281/zenodo.11073129 All raw data from this study can be viewed in the Supporting Data Values file.

## Author contributions

DNE, SW, and JC conceptualized the project and developed methodologies. DNE, SW, KK, WS, LCK, VMN, and YH performed experiments. Data analysis and interpretation was performed by DNE, SW, KK, and JC. KE and MRB provided critical feedback and reagents. KE provided the *Tsc2^fl/fl^* mice. DNE and JC wrote the manuscript. DNE, SW, KK, WS, LCK, VMN, KE, MRB, and JC reviewed and/or revised the manuscript.

## Supplementary Material

Supplemental data

Unedited blot and gel images

Supplemental table 1

Supplemental table 6

Supporting data values

## Version 1 04/08/2025

In-Press Preview

## Version 2 05/08/2025

Electronic publication
