# Supplementary material for: Increased fatty acid delivery by tumor endothelium promotes metastatic outgrowth
Source: JCI Insight. 2025 Apr 8;10(9):e187531. doi: 10.1172/jci.insight.187531 (PMC12128997; doi:10.1172/jci.insight.187531)
Supplement: Supplemental data [file jciinsight-10-187531-s068.pdf]

Supplemental Methods

Immunoblotting: MPMECs were isolated from *Rptor*$^{fl/fl}$ mice and transduced with Cre adenovirus for 16-48 hrs. Ad-CMV-Null was used as a control. To assess VEGF-B activation of mTORC1, cells were serum starved in EBM-2 medium supplemented with 0.2% FBS overnight. A time course was performed on cells treated with 300 ng/mL of recombinant mouse VEGF-B_{186} (R&D Systems #767-VE-010) for 0, 5, 15, or 30 minutes prior to harvest. Pre-cleared lysates were electrophoresed on a SDS-polyacrylamide gel and transferred to nitrocellulose membranes, as previously described (1). The following primary antibodies were used at 1:1000 dilution: Raptor (Cell Signaling Technology (CST) #2280), Rictor (Millipore #05-1471), phospho-S6K1 (T389, CST #9234), S6K1 (CST #9202), phospho-S6RP (S235/236, CST #2211), S6RP (CST #2217), phospho-4EBP1 (T37/46, CST #9459), phospho-4EBP1 (T70, CST #9455), 4EBP1 (CST #9644), phospho-AKT (T308, CST #4056), phospho-AKT (S473, CST #4060), AKT (CST #2920), CLSTN1 (Abcam #ab134130), and β-tubulin (Sigma #T4026). Blots were incubated with secondary antibodies IRDye 680LT goat anti-mouse (1:20,000; LI-COR #925-68020) or IRDye 800 CW goat anti-rabbit (1:10,000; LI-COR #925-32211) and imaged using LI-COR Odyssey.

Permeability assay: $1x10^5$ *Rptor*$^{fl/fl}$ MPMECs were plated onto the inner surface of a gelatin-coated transwell insert with a 0.4 μm pore size. Cells were transduced with Ad-CMV-Null or Ad-CMV-iCre for 16 hours and stimulated with VEGF-B_{186} (300 ng/mL) or VEGF-A (300 ng/mL) for 30 hr in EBM-2 supplemented with 1% BSA free of fatty acids (FFA-BSA). 70 kDa Texas Red-Dextran (1 mg/mL, Thermo #D1830) was placed in the

lower chamber. Ten microliter aliquots of medium were removed from the upper chamber at 0, 10, 30, and 60 min. A transwell without endothelial cells was used to determine maximum permeability intensity. A BioTek Synergy HT plate reader (595 excitation, 615 emission) was used to measure fluorescence intensity of Texas Red-Dextran that passed into the upper chamber. Permeability was calculated as a percent of the empty transwell control.

BODIPY uptake assay: *Rptor*$^{fl/fl}$ MPMECs were transduced with Ad-CMV-Null or Ad-CMV-iCre for 24 hrs. Cells were stimulated with recombinant mouse VEGF-A (300 ng/mL, R&D Systems #493-MV-005), recombinant mouse VEGF-B_{186} (300 ng/mL), mouse VEGFR1/Flt-1 antibody (1 μg/mL, R&D Systems, #AF471), or a combination of anti-VEGFR1 and VEGF-B for 30 hrs. For the combination, cells were pre-incubated with anti-VEGFR1 for 2 hr prior to addition of VEGF-B. Cells were then incubated with 10 μM of BODIPY FL C16 or BODIPY 500/510 C1,C12 (BODIPY-C12, Thermo #D3823) for 3 min. For imaging, cells were fixed in 4% PFA for 10 min and stained with DAPI (Invitrogen #R37606) as directed. Cells were imaged using an Olympus inverted microscope. For flow cytometry, cells were trypsinized and fixed in 4% PFA for 10 min. Flow cytometry was immediately performed as described above.

Immunohistochemistry: Formalin-fixed paraffin-embedded (FFPE) tumor-bearing lungs from WT or *Rptor*^{ECKO} inoculated with E0771 tumor cells were evaluated for phospho-H3 by fluorescent immunohistochemistry, while NDUFS1 was detected by immunohistochemistry, as previously described (1, 2). Antigen retrieval was achieved

using Retreivagen A (BD Biosciences #550524), according to manufacturer's directions. After blocking endogenous peroxidases and blocking with 3% goat serum, tissues were incubated with phospho-H3 (1:100, CST #9701) or NDUFS1 (1:200, Proteintech #12444-1-AP) overnight at 4°C. Phospho-H3 was detected by incubating with an Alexa Fluor 488 secondary antibody (1:500, Invitrogen #A11034), and tissues were mounted in anti-fade with DAPI (Invitrogen #P36941). To detect NDUFS1, sections were incubated with a biotinylated anti-rabbit IgG (1:500; Vector Laboratories, #BA-1000) for 1 hr at room temperature before incubating with streptavidin peroxidase (Vector Laboratories, #SA-5704). Protein was detected using ImmPACT diaminobenzidine (DAB) (Vector Laboratories #SK-4103), prepared according to the manufacturer's instructions. Nuclei were stained with hematoxylin (Vector Laboratories #H-3401), and tissues were mounted using Cytoseal XYL (Epredia #8312-4). At least 2-4 fields of view were obtained using an Olympus inverted microscope. Phospho-H3 positive nuclei and total nuclei were counted using ImageJ software. Total DAB intensity was determined using the deconvolution plug-in within ImageJ software. Percentages and intensities were averaged from all fields of view and normalized to littermate controls.

For H&E images, sections from formalin-fixed, paraffin-embedded lungs were stained with hematoxylin and eosin, as previously described (1). Images were obtained using an Olympus upright inverted microscope.

CLSTN1 immunohistochemistry images: CLSTN1 immunohistochemistry (Gene #ENSG00000171603; Antibody #HPA077705) images in breast lobular carcinoma (3)

(Patient ID #2900), lung squamous cell carcinoma (4) (Patient ID #4488), and melanoma (5) (Patient ID #4229) were obtained from The Human Protein Atlas (6) (www.proteinatlas.org).

Cytotoxicity assay: Cell-mediated cytotoxicity was determined as previously described, with some changes (1). Briefly, E0771-OVA or E0771 parental cells ($1x10^4$ cells per well) were plated in triplicate. OT-I splenocytes were isolated from 8-week-old OT-I female mice and activated with OVA257-264 (SIINFEKL) peptide (1 μg/mL) (Invivogen #vac-sin) for 48 hrs. $CD8^+$ T cells were isolated using mouse CD8a microbeads (Miltenyi Biotec #130-045-201) as previously described (1). E0771-OVA or E0771 parental cells ($1x10^4$ cells per well) were co-cultured with activated CD8+ OT-I T cells ($2x10^3$ cells per well resulting in a 5:1 tumor cell to T cell ratio) in in 0 or 50 μM palmitate-BSA for 48 hrs. Cytotoxicity was measured using the CyQUANT lactate dehydrogenase (LDH) cytotoxicity assay (Invitrogen #C20300), according to manufacturer's instructions and as previously indicated (1). Cytotoxicity was calculated as a percent of maximum LDH release upon cell lysis, accounting for spontaneous cell death under the respective culture conditions.

Sphere invasion assay: Invasion of E0771 cells was performed as previously described (7, 8), with some changes. Briefly, E0771 cells ($2.5x10^4$ cells/mL) were cultured as 20μL hanging drops for 72 hrs in DMEM supplemented with 10% FBS. Spheres were then embedded in 200 μL of a mix of growth factor reduced Matrigel (50%, Corning #354230) and type I rat tail collagen (0.5 mg/mL, Gibco #A1048301)

supplemented with 0 or 50μM palmitate-BSA. Spheroids were incubated at 37°C for 30 min to allow Matrigel to solidify, followed by submersion in 1 mL of warm cell culture media supplemented with 0 or 50μM palmitate-BSA. Bright field images were taken at 48 hr using an inverted microscope. Invasive area was determined from 8-10 fields of view using ImageJ software, corrected for original sphere area.

Quantitative real-time PCR: RNA was collected from *Rptor*$^{fl/fl}$ or *Tsc2*$^{fl/fl}$ MPMECs transduced with Ad-CMV-Null or Ad-CMV-iCre, as described above. Following the manufacturer's directions, cDNA was generated using the iScript cDNA synthesis kit (Bio-Rad #1708891). Using the primers defined in Supplemental Table 4, PCR amplification was performed in triplicate using the StepOnePlus (Applied Biosystems) as previously described (1). The $\Delta\Delta C_t$ quantitation method was used.

References

1. Edwards DN, et al. Selective glutamine metabolism inhibition in tumor cells improves anti-tumor T lymphocyte activity in triple-negative breast cancer. *J Clin Invest*. 2021;131(4):e140100.

2. Edwards D, et al. The receptor tyrosine kinase EphA2 promotes glutamine metabolism in tumors by activating the transcriptional coactivators YAP and TAZ. *Sci Signal*. 2017;10(508):eaan4667.

3. The Human Protein Atlas. CLSTN1 IHC images in breast cancer [Internet]. *http://www.proteinatlas.org/ENSG00000171603-CLSTN1/pathology/breast+cancer#img*. Accessed August 9, 2023.

4. The Human Protein Atlas. CLSTN1 IHC images in lung cancer [Internet]. *http://www.proteinatlas.org/ENSG00000171603-CLSTN1/pathology/lung+cancer#img*. Accessed August 9, 2023.

5. The Human Protein Atlas. CLSTN1 IHC images in melanoma [Internet]. *http://proteinatlas.org/ENSG00000171603-CLSTN1/pathology/melanoma#img*. Accessed August 9, 2023.

6. Uhlén M, et al. Tissue-based map of the human proteome. *Science*. 2015;347(6220):1260419.

7. Reuten R, et al. Basement membrane stiffness determines metastases formation. *Nat Mater*. 2021;20(6):892–903.

8. Berens EB, et al. A cancer cell spheroid assay to assess invasion in a 3D setting. *J Vis Exp*. 2015;20(105):53409.

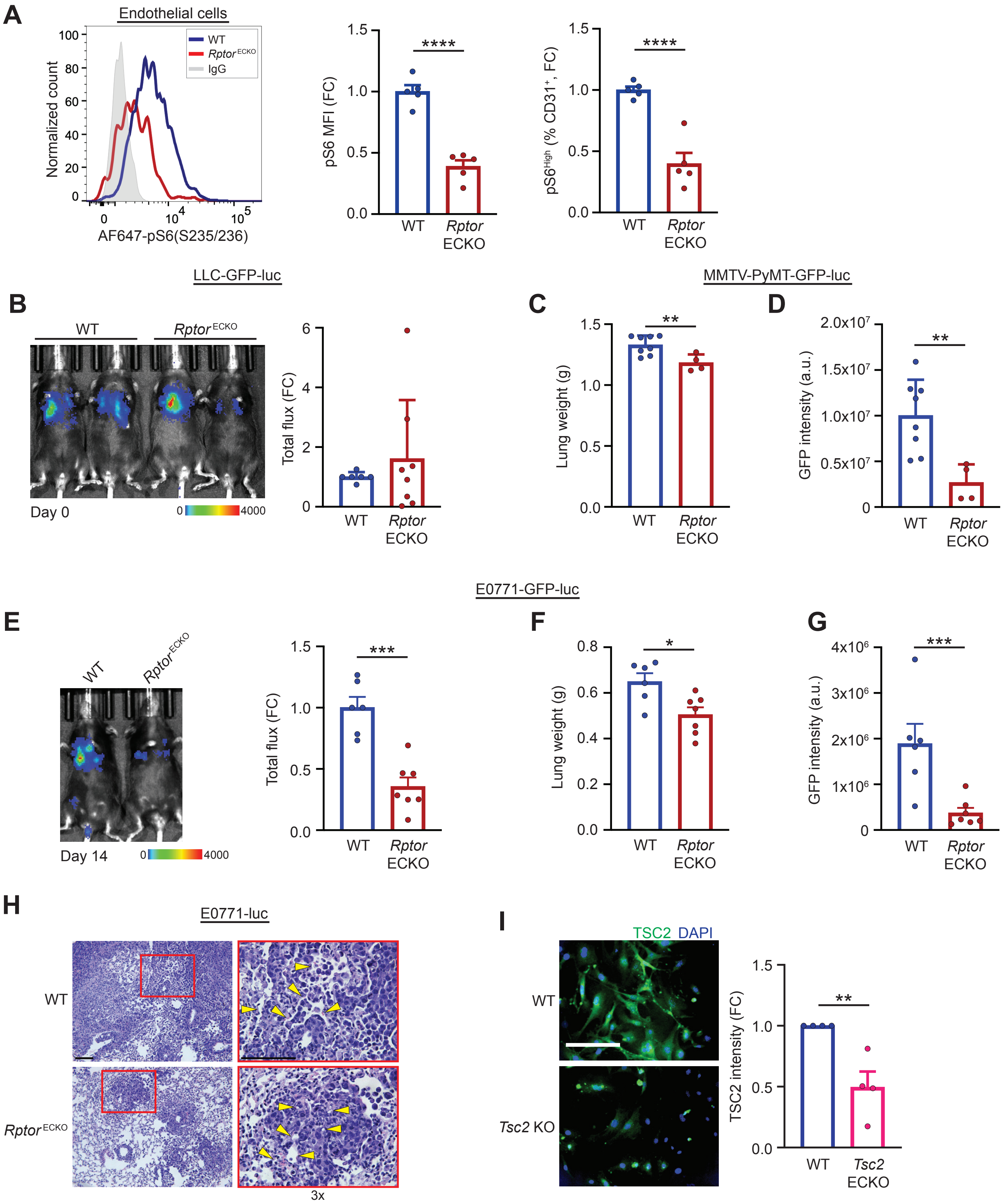
A
Endothelial cells
WT
Rptor ECKO
IgG
Normalized count
100
80
60
40
20
0
0
10^4
10^5
AF647-pS6(S235/236)
pS6 MFI (FC)
1.5
1.0
0.5
0.0
****
WT
Rptor ECKO
pS6High (% CD31+, FC)
1.5
1.0
0.5
0.0
****
WT
Rptor ECKO
LLC-GFP-luc
MMTV-PyMT-GFP-luc
B
WT
Rptor ECKO
Day 0
0
4000
Total flux (FC)
6
4
2
0
WT
Rptor ECKO
C
Lung weight (g)
1.5
1.0
0.5
0.0
**
WT
Rptor ECKO
D
GFP intensity (a.u.)
2.0x10^7
1.5x10^7
1.0x10^7
0.5x10^7
0
**
WT
Rptor ECKO
E0771-GFP-luc
E
WT
Rptor ECKO
Day 14
0
4000
Total flux (FC)
1.5
1.0
0.5
0.0
***
WT
Rptor ECKO
F
Lung weight (g)
0.8
0.6
0.4
0.2
0.0
*
WT
Rptor ECKO
G
GFP intensity (a.u.)
4x10^6
3x10^6
2x10^6
1x10^6
0
***
WT
Rptor ECKO
H
E0771-luc
WT
Rptor ECKO
3x
I
TSC2 DAPI
WT
Tsc2 KO
TSC2 intensity (FC)
1.5
1.0
0.5
0.0
**
WT
Tsc2 ECKO

Supplemental Figure 1. Loss of Raptor/mTORC1 in endothelium reduces metastatic outgrowth of cancer cells in the lung. (A) WT (n=5) or *Rptor*^{ECKO} (n=7) female mice were treated with tamoxifen for 5 days and lungs were harvested 1 week following last treatment. Phospho-S6 (pS6) (S235/236) median fluorescence intensity (MFI) and percentage of pS6^{High} CD31^{+} endothelial cells was determined by flow cytometry, normalized to WT littermate controls. Unpaired t-test: pS6 MFI, $p=3.23\times10^{-5}$; pS6^{High}, $p=2.04\times10^{-4}$. **(B)** Representative bioluminescence images from Day 0 of LLC-GFP-luc inoculated WT or *Rptor*^{ECKO} male mice. Scale bar shows counts. Total radiance flux was normalized to WT controls. WT (n=6), *Rptor*^{ECKO} (n=8). Unpaired t-test, p=0.472. **(C-D)** WT (n=8) or *Rptor*^{ECKO} (n=4) female mice were inoculated with MMTV-PyMT-GFP-luc cells, followed by tamoxifen treatment starting on day 4. Tumors were harvested on day 14. **(C)** Lung weights were recorded in grams (g) at harvest and **(D)** GFP intensity was calculated as arbitrary units (a.u.). Unpaired t-test, $p=9.75\times10^{-3}$ for lung weights and $p=6.30\times10^{-3}$ for GFP intensity. **(E-G)** WT (n=6) or *Rptor*^{ECKO} (n=7) female mice were inoculated with E0771-GFP-luc cells as described in (B). **(E)** Representative bioluminescence images are shown from day 14. Scale bar shows counts. Total radiance flux was normalized to WT controls. Unpaired t-test, $p=1.65\times10^{-3}$. Tumors were harvested on day 18. **(F)** Lung weights were recorded in grams (g) at harvest and **(G)** GFP intensity was calculated. Unpaired t-test, p=0.0131 for lung weights and $p=3.97\times10^{-3}$ for GFP intensity. **(H)** WT (n=9) or *Rptor*^{ECKO} (n=8) female mice were inoculated with E0771-luc cells as described in (E-G). Representative lung H&E images are shown, with 3x magnified region indicated by the red box. Blood vessels are denoted by yellow arrows. Scale bars are 100 μm. **(I)** Primary microvascular endothelial cells were isolated from *Tsc2*$^{fl/fl}$ mice were transduced with control (WT) or cre-expressing (*Tsc2* KO) adenovirus. Immunofluorescence was used to confirm *Tsc2* deletion (n=4 per group). Representative images of TSC2 (green) are shown. Nuclei are stained with DAPI (blue). Scale bar is 100 μm. Fluorescence intensity was normalized to WT control. Unpaired t-test, $p=8.11\times10^{-3}$. *p<0.05, **p<0.01, ***p<0.005, ****p<0.001. Related to Figure 1.

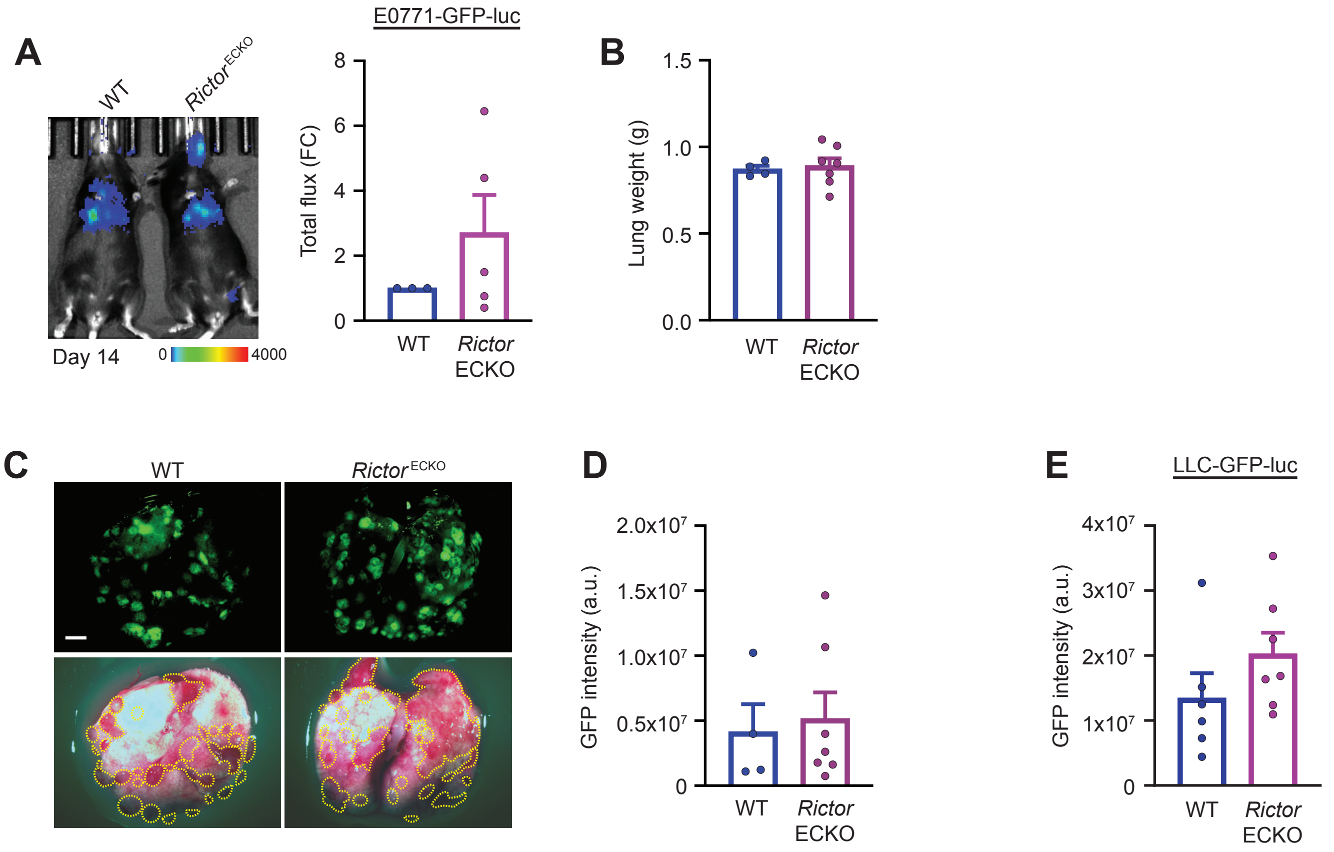

Supplemental Figure 2. Loss of Rictor/mTORC2 in endothelium does not significantly alter metastatic outgrowth of cancer cells in the lung. (A-D) WT (n=3) or *Rictor*^{ECKO} (n=5) female mice were inoculated with E0771-GFP-luc cells and treated with tamoxifen as described in Figure 1. **(A)** Representative bioluminescence images from day 14 are shown. Scale bar shows counts. Total radiance flux was normalized to WT controls. Unpaired t-test, p=0.318. **(B)** Tumors were harvested on day 18, and lung weights were recorded in grams (g). WT (n=4), *Rictor*^{ECKO} (n=7). Unpaired t-test, p=.767. **(C)** Representative GFP (top) and gross (bottom) lung images are shown. Scale bar is 5 mm. Visible tumor area is outlined by yellow line. **(D)** GFP intensity was calculated as arbitrary units (a.u.). WT (n=4), *Rictor*^{ECKO} (n=7). Unpaired t-test, p=0.753. **(E)** WT (n=6) or *Rictor*^{ECKO} (n=7) male mice were inoculated with LLC-GFP-luc cells as described above. Tumors were harvested on day 18. Lung weights were recorded. Unpaired t-test, p=0.204. Related to Figure 1.

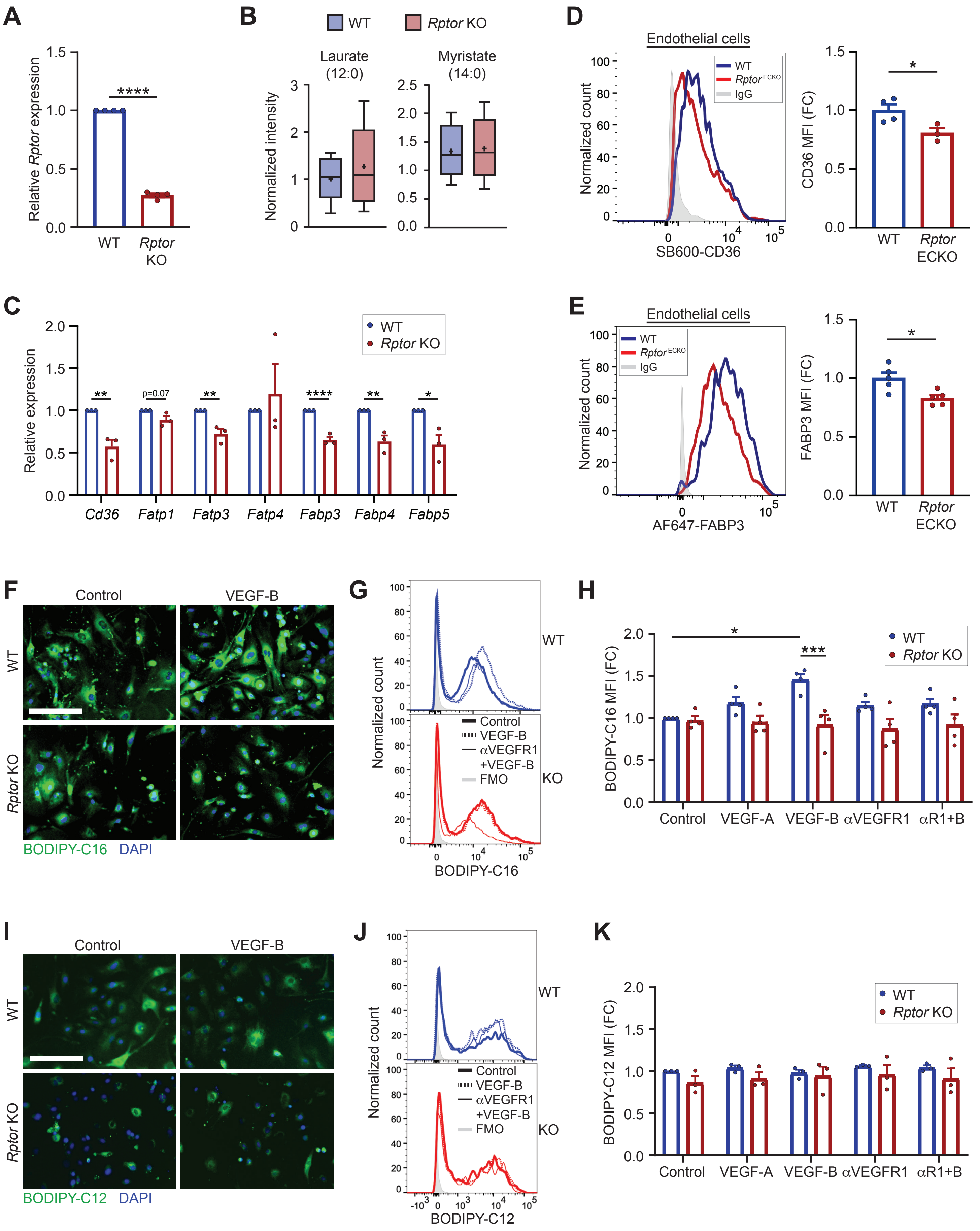
A
Relative Rptor expression
WT
Rptor KO
B
WT
Rptor KO
Laurate (12:0)
Myristate (14:0)
Normalized intensity
C
Relative expression
Cd36
Fatp1
Fatp3
Fatp4
Fabp3
Fabp4
Fabp5
p=0.07
D
Endothelial cells
Normalized count
SB600-CD36
IgG
CD36 MFI (FC)
Rptor ECKO
E
AF647-FABP3
FABP3 MFI (FC)
F
Control
VEGF-B
BODIPY-C16
DAPI
G
Control
αVEGFR1 +VEGF-B
FMO
KO
H
BODIPY-C16 MFI (FC)
VEGF-A
αVEGFR1
αR1+B
I
BODIPY-C12
J
K
BODIPY-C12 MFI (FC)

Supplemental Figure 3. Targeting Raptor/mTORC1 reduces fatty acid long-chain fatty acid content in endothelial cells. **(A-B)** Metabolomics was performed on primary microvascular endothelial cells isolated from *Rptor*$^{fl/fl}$ mice transduced with control (WT) or cre recombinase (KO) adenoviruses, as described in Figure 2. **(A)** Rptor deletion was confirmed by reduced *Rptor* RNA expression by qRT-PCR (n=4 per group). Unpaired t-test, $p=6.19x10^{-9}$. **(B)** Normalized intensities (log_2+1) of representative medium chain fatty acid (MCFA) metabolites (n=6 per group). The median along with the 25th and 75th percentile hinges are indicated within the box. The whiskers indicate minimum and maximum values within each group. The mean is shown as a plus sign ("+"). Welch's t-test; 12:0, p=0.533; 14:0, p=0.879. **(C)** Relative expression of fatty acid transporters and fatty acid binding proteins was determined by qRT-PCR (n=4 per group). Unpaired t-test, *Cd36*, p=0.00619; *Slc27a1*/*Fatp1*, p=0.0685; *Slc27a3*/*Fatp3*, p=0.00619; *Slc27a4*/*Fatp4*, p=0.610; *Fabp3*, p=0.000741; *Fabp4*, p=0.00672; *Fabp5*, p=0.0236. **(D-E)** WT or *Rptor*^{ECKO} female mice were treated with tamoxifen for 5 days**,** and lungs were harvested 1 week following last treatment. Median fluorescence intensity (MFI) of **(D)** CD36 and **(E)** FABP3 was determined by flow cytometry in $CD31^+$ endothelial cells, normalized to WT littermate controls. CD36: WT (n=4), *Rptor*^{ECKO} (n=3); FABP3: WT (n=5), *Rptor*^{ECKO} (n=5). Unpaired t-test; CD36, p=0.0392; FABP3, p=0.0140. **(F-K)** Primary microvascular endothelial cells were isolated from *Rptor*$^{fl/fl}$ mice, and deletion was carried out by transduction with control (WT) or cre-expressing (KO) adenovirus for 48 hours. Cells were then treated with VEGF-B (300 ng/mL), VEGF-A (300 ng/mL), anti-VEGFR1 (1 μg/mL), or anti-VEGFR1+VEGF-B (αR1+B) for 30 hours in free fatty acid BSA (FFA-BSA) supplemented media. Control cells were untreated. Cells were immediately incubated with **(F-H)** BODIPY FL C16 (BODIPY-C16, 20 μg/mL) or **(I-K)** BODIPY-C12 (20 μg/mL) for 3 min. **(F)** Representative images of BODIPY-C16 (green) are shown. Nuclei were stained with DAPI (blue). Scale bar is 100 μm. **(G-H)** BODIPY-C16 fluorescence was analyzed by flow cytometry (n=4 per group). **(G)** Representative histogram plots of Control, VEGF-B, and anti-VEGFR1+VEGF-B groups are shown. **(H)** BODIPY-C16 median fluorescence intensity (MFI) was normalized to WT Control. Two-way ANOVA ($p=1.56x10^{-5}$) with Tukey's post hoc. **(I)** Representative images of BODIPY-C12 (green) are shown. Nuclei were stained with DAPI (blue). Scale bar is 100 μm. **(J-K)** BODIPY-C12 fluorescence was analyzed by flow cytometry (n=3 per group). **(J)** Representative histogram plots of Control, VEGF-B, and anti-VEGFR1+VEGF-B are shown. **(K)** BODIPY-C12 MFI was normalized to WT Control. Two-way ANOVA (p=0.0339) with Tukey's post hoc, which revealed no significant comparisons. *p<0.05, **p<0.01, ***p<0.005, ****p<0.001. Related to Figure 2 and Supplemental Table 1.

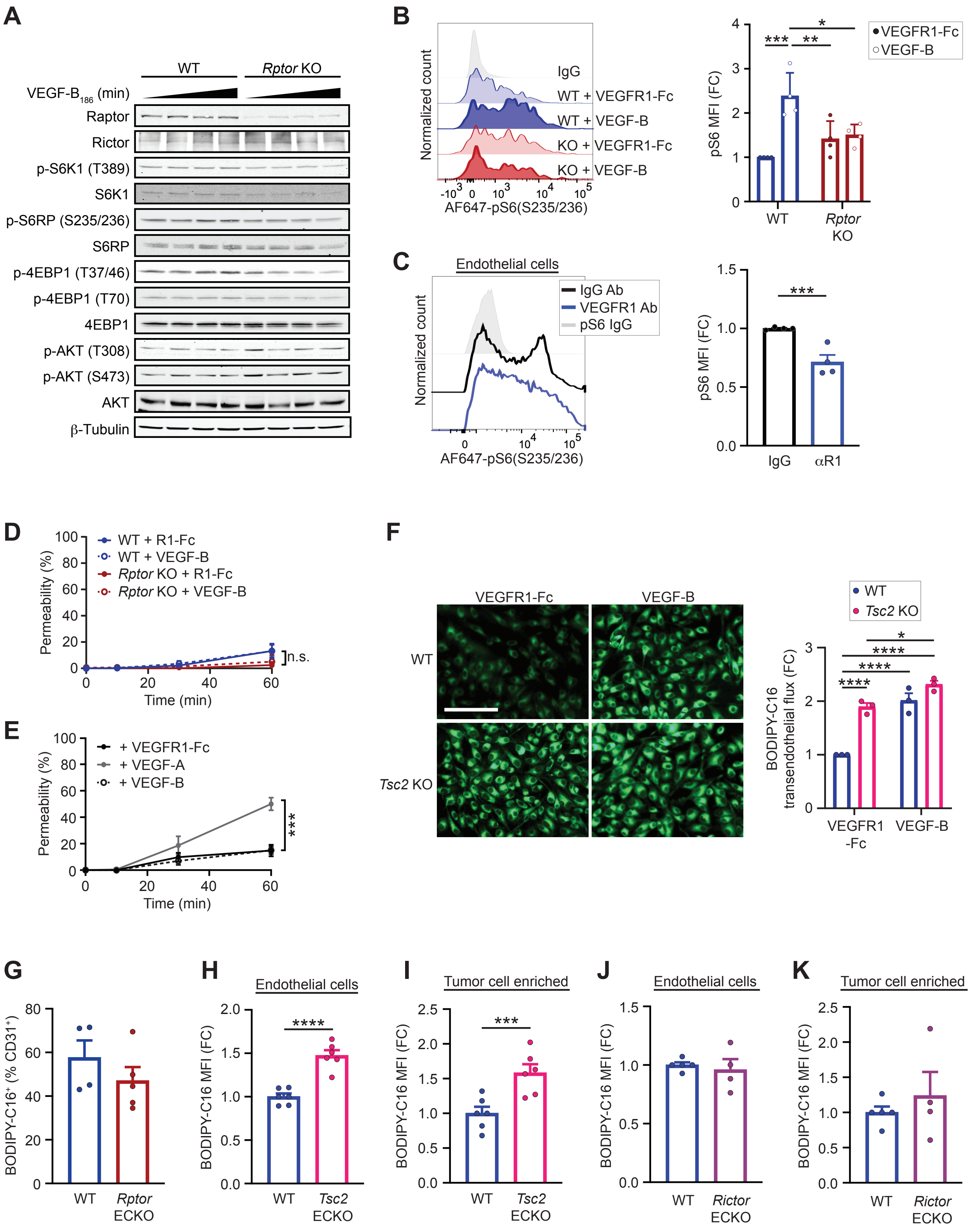
A
WT
Rptor KO
VEGF-B186 (min)
Raptor
Rictor
p-S6K1 (T389)
S6K1
p-S6RP (S235/236)
S6RP
p-4EBP1 (T37/46)
p-4EBP1 (T70)
4EBP1
p-AKT (T308)
p-AKT (S473)
AKT
β-Tubulin
B
Normalized count
IgG
WT + VEGFR1-Fc
WT + VEGF-B
KO + VEGFR1-Fc
KO + VEGF-B
AF647-pS6(S235/236)
pS6 MFI (FC)
VEGFR1-Fc
VEGF-B
WT
Rptor KO
C
Endothelial cells
IgG Ab
VEGFR1 Ab
pS6 IgG
Normalized count
AF647-pS6(S235/236)
pS6 MFI (FC)
IgG
αR1
D
WT + R1-Fc
WT + VEGF-B
Rptor KO + R1-Fc
Rptor KO + VEGF-B
Permeability (%)
n.s.
Time (min)
E
+ VEGFR1-Fc
+ VEGF-A
+ VEGF-B
Permeability (%)
Time (min)
F
VEGFR1-Fc
VEGF-B
WT
Tsc2 KO
BODIPY-C16 transendothelial flux (FC)
VEGFR1 -Fc
G
BODIPY-C16+ (% CD31+)
Rptor ECKO
H
Endothelial cells
BODIPY-C16 MFI (FC)
Tsc2 ECKO
I
Tumor cell enriched
J
Rictor ECKO
K

Supplemental Figure 4. Endothelial Raptor/mTORC1 promotes transendothelial transport of fatty acids in response to VEGF-B. (A-B) Primary microvascular endothelial cells were isolated from *Rptor*$^{fl/fl}$ mice, and deletion was carried out by transduction with control (WT) or cre-expressing (KO) adenovirus for 48 hrs. **(A)** Cells were serum starved overnight and stimulated with VEGF-B (300 ng/mL) for 0, 5, 15, or 30 min. Lysates were harvested for signaling analysis by immunoblotting. Blot is representative of two independent experiments. **(B)** Cells were serum starved as described in (A) and treated with VEGFR1-Fc (1 μg/mL) or VEGF-B (300 ng/mL) for 30 hr. Cells were analyzed for pS6 (S235/236) by flow cytometry (n=4 per group). Two-way ANOVA (p=0.00316) with Tukey's post hoc. **(C)** Mice bearing E0771-GFP-luc lung metastases were treated with anti-VEGFR1 antibody or IgG control (n=4 per group). CD31^{+} endothelial cells from harvested tumors were analyzed for pS6 as in (B). Unpaired t-test, p=0.003259. **(D-E)** Endothelial permeability assay was performed by detecting diffusion of Texas Red (TR)-Dextran across a transwell coated with a confluent top layer of WT or *Rptor* KO primary microvascular endothelial cells. Cells were treated with VEGFR1-Fc, VEGF-B, or VEGF-A for 30 hrs immediately before performing the assay. Media was removed from the upper chamber at 0, 10, 30, and 60 min and fluorescence was analyzed on a plate reader (n=3-4 per group). Two-way ANOVA (D: p=0.2809; E: p=2.949×10^{-6}) with Tukey's post hoc. **(F)** Transendothelial transport assay of WT or *Tsc2* KO primary vascular endothelial cells (n=3 per group), as described in Figure 3. Representative images of BODIPY-C16 (green) in LLC tumor cells. Scale bar is 100 μm. BODIPY-C16 intensity was normalized to WT + VEGFR1-Fc control. Two-way ANOVA (p=8.04×10^{-3}) with Tukey's post hoc. **(G)** WT (n=4) or *Rptor*^{ECKO} (n=5) male mice were inoculated with LLC cells and BODIPY-C16 fluorescence was determined by flow cytometry as described in Figure 3E-F. Percentage (%) of BODIPY^{+} CD31^{+} endothelial cells are shown. Unpaired t-test, p=0.3194. **(H-I)** WT (n=6) or *Tsc2*^{ECKO} (n=6) female mice were inoculated with E0771-luc cells and injected with BODIPY FL C16 (BODIPY-C16), as described in Figure 3. BODIPY-C16 median fluorescence intensity (MFI) was determined by flow cytometry in **(H)** CD45^{-}CD31^{+} endothelial cells and **(I)** CD45^{-}CD31^{-}FSC^{hi} tumor-cell enriched populations and normalized to WT controls. Representative histograms are shown. Unpaired t-test; CD31^{+}, p=6.26×10^{-5}; tumor-cell enriched, p=3.90×10^{-3}. **(J-K)** WT (n=5) or *Rictor*^{ECKO} (n=4) male mice were inoculated with LLC cells and injected with BODIPY FL C16 (BODIPY-C16), as described above. BODIPY-C16 MFI was determined by flow cytometry in **(J)** CD45^{-}CD31^{+} endothelial cells and **(K)** CD45^{-}CD31^{-}FSC^{hi} tumor-cell enriched populations and normalized to WT controls. Representative histograms are shown. Unpaired t-test; CD31^{+}, p=0.577; tumor-cell enriched, p=0.376. *p<0.05, **p<0.01, ***p<0.005, ****p<0.001. Related to Figure 3.

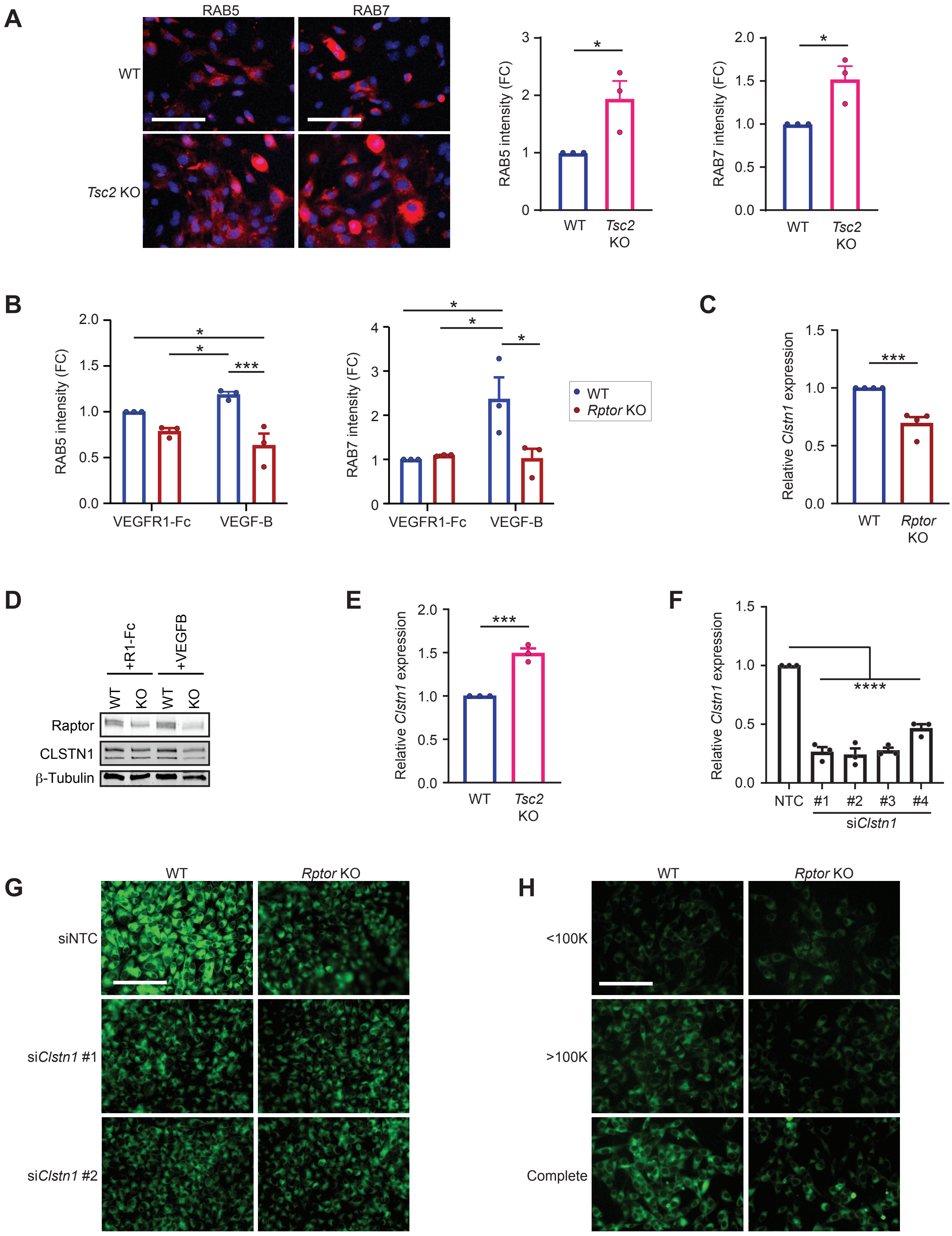
A
RAB5
RAB7
WT
Tsc2 KO
RAB5 intensity (FC)
RAB7 intensity (FC)
WT
Tsc2 KO
B
RAB5 intensity (FC)
RAB7 intensity (FC)
VEGFR1-Fc
VEGF-B
WT
Rptor KO
C
Relative Clstn1 expression
WT
Rptor KO
D
+R1-Fc
+VEGFB
WT
KO
Raptor
CLSTN1
β-Tubulin
E
Relative Clstn1 expression
WT
Tsc2 KO
F
Relative Clstn1 expression
NTC
#1
#2
#3
#4
siClstn1
G
WT
Rptor KO
siNTC
siClstn1 #1
siClstn1 #2
H
WT
Rptor KO
<100K
>100K
Complete

Supplemental Figure 5. mTORC1 increases RAB endosomes and regulates CLSTN1 expression to support transendothelial transport of fatty acids in endothelial cells. (A) Immunofluorescence of RAB5 (left) or RAB7 (right) was performed on WT or *Tsc2* KO primary microvascular endothelial cells cultured in complete endothelial cell media (n=3 per group). Representative immunofluorescence images of RAB5 and RAB7 are shown (both red). Nuclei are stained with DAPI (blue). Scale bars are 100 μm. RAB5 or RAB7 intensities were normalized to WT controls. Unpaired t-test; RAB5, p=0.0371; RAB7, p=0.0255. **(B)** WT or *Rptor* KO primary microvascular endothelial cells cultured in basal media supplemented with FFA-BSA were stimulated with VEGFR1-Fc (1 μg/mL) or VEGF-B (300 ng/mL) for 30 hours (n=3 per group). Immunofluorescence and intensities were performed as in (A). Two-way ANOVA (RAB5, p=0.0372; RAB7, p=0.0283) with Tukey's post hoc. **(C-D)** WT or *Rptor* KO primary microvascular endothelial cells were assessed for *Clstn1* expression by **(C)** qRT-PCR (n=4 per group) and **(D)** immunoblotting (image representative of 3 independent experiments). Unpaired t-test: *Clstn1* expression, $p=1.27 \times 10^{-3}$. **(E)** *Clstn1* expression was analyzed in WT or *Tsc2* KO primary microvascular endothelial cells by qRT-PCR (n=3 per group). Unpaired t-test, $p=9.96 \times 10^{-4}$. **(F)** Knockdown of *Clstn1* using siRNA was confirmed by reduced *Clstn1* expression in *Rptor*$^{fl/fl}$ primary microvascular endothelial cells treated with control adenovirus (n=3 per group). One-way ANOVA ($p=1.84 \times 10^{-7}$) with Dunnett's post hoc. **(G)** Representative images of LLC tumor cells from transendothelial transport of BODIPY-C16 (green) in non-targeting control (NTC) or *Clstn1* knockdown endothelial cells from Figure 4. Scale bar is 100 μm. **(H)** Representative images of LLC tumor cells cultured in small (<100 kDa) or large (>100 kDa) fractionated endothelial cell conditioned media from Figure 4. Images from complete conditioned media are also shown. Scale bar is 100 μm. *p<0.05, ***p<0.005, ****p<0.001. Related to Figure 4.

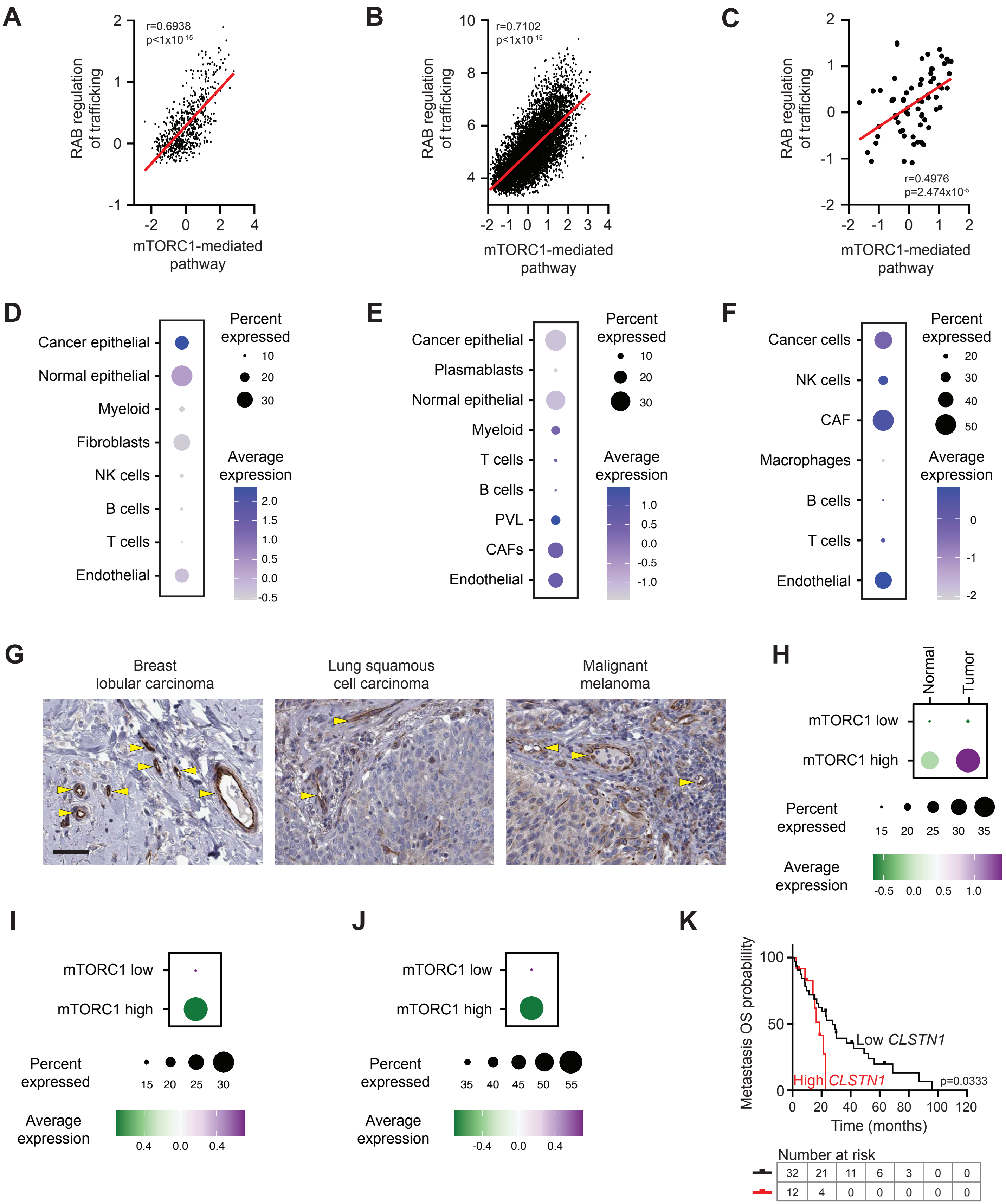
A
r=0.6938
p<1x10-15
RAB regulation of trafficking
mTORC1-mediated pathway
B
r=0.7102
p<1x10-15
RAB regulation of trafficking
mTORC1-mediated pathway
C
r=0.4976
p=2.474x10-5
RAB regulation of trafficking
mTORC1-mediated pathway
D
Cancer epithelial
Normal epithelial
Myeloid
Fibroblasts
NK cells
B cells
T cells
Endothelial
Percent expressed
10
20
30
Average expression
2.0
1.5
1.0
0.5
0.0
-0.5
E
Cancer epithelial
Plasmablasts
Normal epithelial
Myeloid
T cells
B cells
PVL
CAFs
Endothelial
Percent expressed
10
20
30
Average expression
1.0
0.5
0.0
-0.5
-1.0
F
Cancer cells
NK cells
CAF
Macrophages
B cells
T cells
Endothelial
Percent expressed
20
30
40
50
Average expression
0
-1
-2
G
Breast lobular carcinoma
Lung squamous cell carcinoma
Malignant melanoma
H
Normal
Tumor
mTORC1 low
mTORC1 high
Percent expressed
15 20 25 30 35
Average expression
-0.5 0.0 0.5 1.0
I
mTORC1 low
mTORC1 high
Percent expressed
15 20 25 30
Average expression
0.4 0.0 0.4
J
mTORC1 low
mTORC1 high
Percent expressed
35 40 45 50 55
Average expression
-0.4 0.0 0.4
K
Metastasis OS probability
Low CLSTN1
High CLSTN1
p=0.0333
Time (months)
Number at risk
32 21 11 6 3 0 0
12 4 0 0 0 0 0

Supplemental Figure 6. mTORC1 signaling correlates with RAB trafficking and *CLSTN1* expression in human tumor-associated endothelial cells. (A-C) mTORC1 signaling positively correlates with RAB regulation of trafficking gene set in endothelial cells from the **(A)** lung adenocarcinoma (Bischoff, et al.), **(B)** breast cancer (GSE176078), and **(C)** melanoma (GSE72056) single-cell RNA-seq datasets. The x and y axis represents z-scores of ssGSEA enrichment of indicated gene sets. **(D-E)** Dot plots of *CLSTN1* expression in cell populations from **(D)** lung adenocarcinoma, **(E)** breast cancer, and **(F)** melanoma datasets above. **(G)** Immunohistochemistry of CLSTN1 from patient samples showing strong staining in vascular endothelial regions, denoted by yellow arrows. Images were acquired from the Human Protein Atlas. **(H)** Dot plot of *CLSTN1* expression in normal or tumor-associated endothelial cells, stratified based on mTORC1 signaling ssGSEA enrichrment scores, in the lung adenocarcinoma dataset used above. **(I-J)** Dot plot of *CLSTN1* expression in tumor-associated endothelial cells in **(I)** breast cancer and **(J)** melanoma datasets described above. mTORC1 signaling stratification was performed as described in (H). **(K)** Overall survival (OS) of metastatic breast cancer patients stratified by low (quartiles 1-3, black) or high (quartile 4, red) *CLSTN1*:*PECAM1* ratio. Data collected from the AURORA dataset (GSE209998). Hazard ratio is 2.114 (95% CI: 0.8157 - 5.481). Related to Figure 4 and Supplemental Table 6.

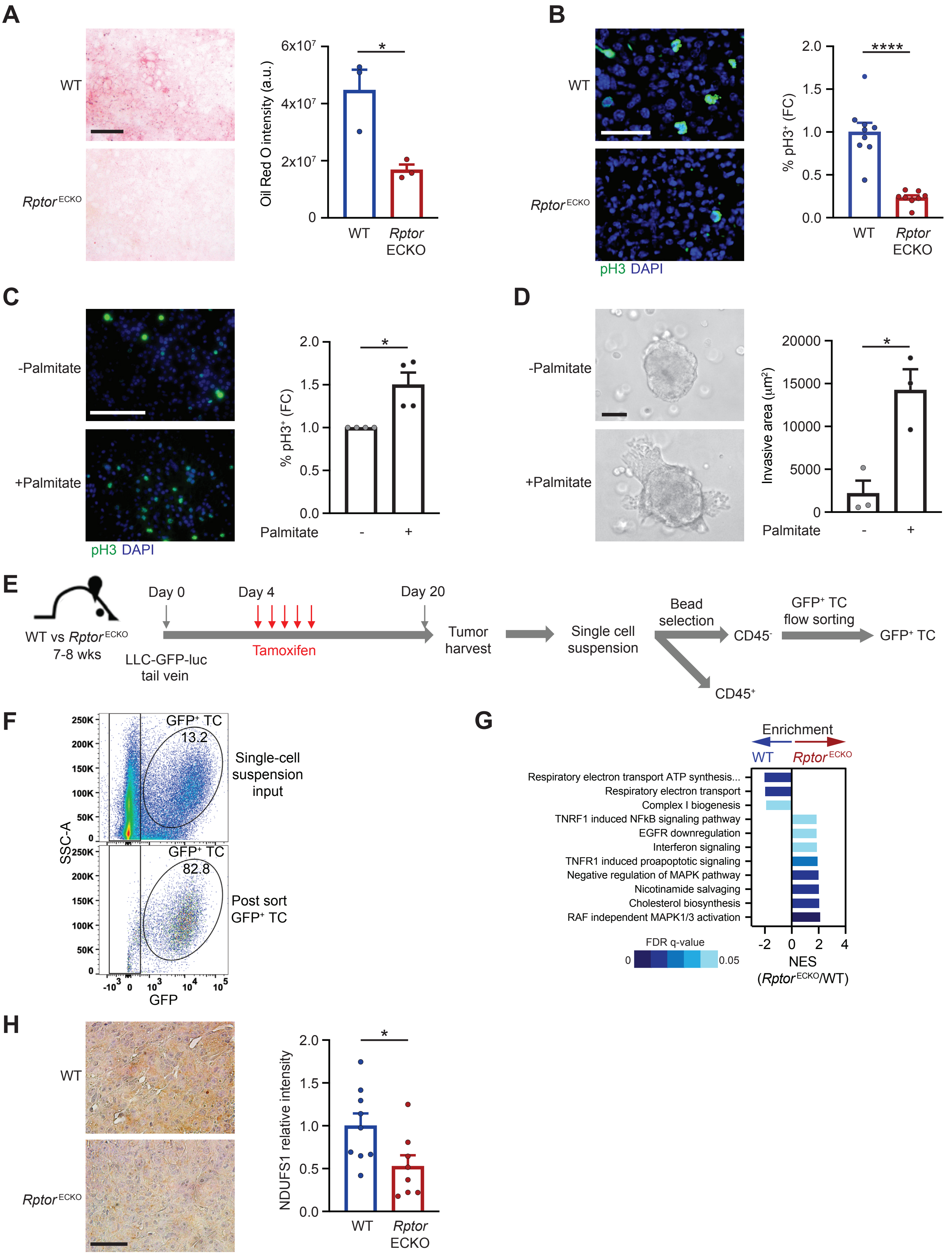
A
WT
Rptor ECKO
Oil Red O intensity (a.u.)
6x10^7
4x10^7
2x10^7
0
*
WT
Rptor ECKO
B
WT
Rptor ECKO
pH3 DAPI
% pH3+ (FC)
2.0
1.5
1.0
0.5
0.0
****
WT
Rptor ECKO
C
-Palmitate
+Palmitate
pH3 DAPI
% pH3+ (FC)
*
Palmitate
-
+
D
-Palmitate
+Palmitate
Invasive area (µm2)
20000
15000
10000
5000
0
*
Palmitate
-
+
E
WT vs Rptor ECKO
7-8 wks
Day 0
LLC-GFP-luc
tail vein
Day 4
Tamoxifen
Day 20
Tumor harvest
Single cell suspension
Bead selection
CD45-
CD45+
GFP+ TC flow sorting
GFP+ TC
F
SSC-A
250K
200K
150K
100K
50K
0
GFP+ TC
13.2
Single-cell suspension input
GFP+ TC
82.8
Post sort GFP+ TC
GFP
G
Enrichment
WT
Rptor ECKO
Respiratory electron transport ATP synthesis...
Respiratory electron transport
Complex I biogenesis
TNRF1 induced NFkB signaling pathway
EGFR downregulation
Interferon signaling
TNFR1 induced proapoptotic signaling
Negative regulation of MAPK pathway
Nicotinamide salvaging
Cholesterol biosynthesis
RAF independent MAPK1/3 activation
FDR q-value
0
0.05
-2
0
2
4
NES
(Rptor ECKO/WT)
H
WT
Rptor ECKO
NDUFS1 relative intensity
2.0
1.5
1.0
0.5
0.0
*
WT
Rptor ECKO

Supplemental Figure 7. Endothelial mTORC1 promotes tumor cell invasion and proliferation. (A) WT or *Rptor*^{ECKO} male mice were inoculated with LLC-GFP-luc cells, as in Figure 1A. Harvested lung were cryopreserved, and sections were stained with Oil Red O (n=3 per group). Representative images are shown. Scale bar is 50μm. Oil Red O intensities are shown. Unpaired t-test, p=0.0204. **(B)** WT (n=9) or *Rptor*^{ECKO} (n=8) female mice were inoculated with E0771-luc cells as in Figure 1. Fluorescent immunohistochemistry for phospho-H3 (pH3, green) was performed, and percent positive nuclei was determined. Nuclei are stained with DAPI (blue). Representative images are shown. Scale bar is 50 μm. Unpaired t-test, $p=8.91x10^{-6}$. **(C-D)** E0771 cells were cultured in the absence or presence of 50 μM of palmitate-BSA conjugate for 48 hrs. **(C)** Immunofluorescence of phospho-H3 (pH3, green) was performed (n=4 per group). Representative images are shown. Nuclei are stained with DAPI (blue). Scale bar is 100 μm. Percentages of positive cells are shown. Unpaired t-test, p=0.0128. **(D)** 3D sphere invasion assay was performed (n=3 per group). Mean invasive area ($μm^2$) of spheroids is shown. Unpaired t-test, p=0.0138. **(E)** Schematic of GFP^+ tumor cell sorting of WT and *Rptor*^{ECKO} lung metastatic tumors. **(F)** Flow cytometry confirmed enrichment of GFP^+ tumor cells (TC) after sorting. Representative data are shown. **(G)** Sorted GFP^+ tumor cells from WT or *Rptor*^{ECKO} lung metastatic tumors were analyzed by RNA-seq (n=4 per group) and differentially expressed genes were identified. GSEA was performed to identify pathway enrichment. Normalized enrichment score (NES) and false discovery rate (FDR) q-values are indicated. **(H)** Immunohistochemistry of NDUFS1 (brown) in WT (n=9) or *Rptor*^{ECKO} (n=8) metastatic tumors formed from E0771-luc cell inoculation as in Figure 1A. Nuclei were stained with hematoxylin (blue). Scale bar is 50 μm. Intensities were normalized to controls. Unpaired t-test, p=0.0290. *p<0.05, ****p<0.001. Related to Figure 5.

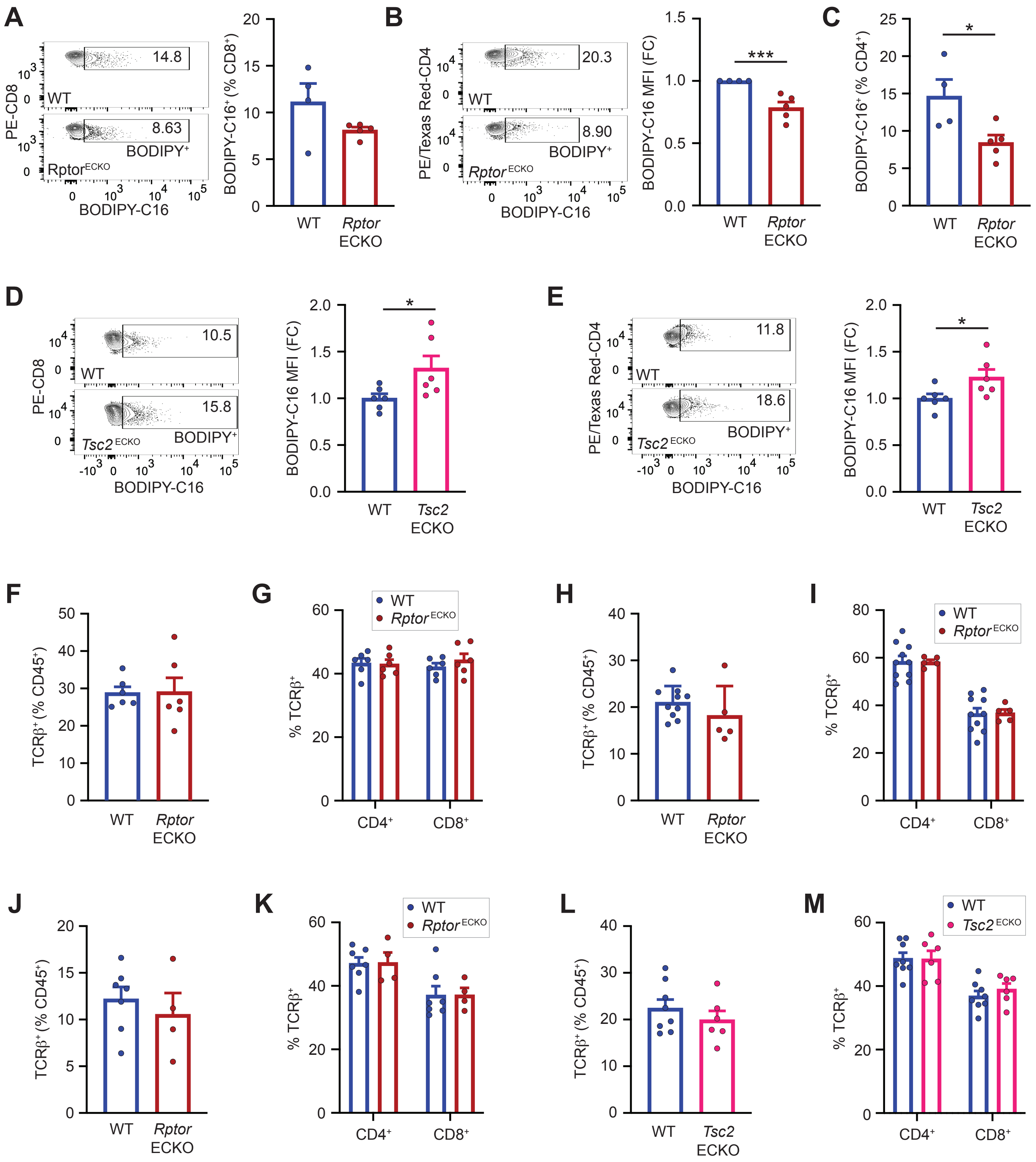

A
PE-CD8
14.8
8.63
WT
Rptor ECKO
BODIPY+
BODIPY-C16
BODIPY-C16+ (% CD8+)
WT
Rptor ECKO
B
PE/Texas Red-CD4
20.3
8.90
WT
Rptor ECKO
BODIPY+
BODIPY-C16
BODIPY-C16 MFI (FC)
***
WT
Rptor ECKO
C
BODIPY-C16+ (% CD4+)
*
WT
Rptor ECKO
D
PE-CD8
10.5
15.8
WT
Tsc2 ECKO
BODIPY+
BODIPY-C16
BODIPY-C16 MFI (FC)
*
WT
Tsc2 ECKO
E
PE/Texas Red-CD4
11.8
18.6
WT
Tsc2 ECKO
BODIPY+
BODIPY-C16
BODIPY-C16 MFI (FC)
*
WT
Tsc2 ECKO
F
TCRβ+ (% CD45+)
WT
Rptor ECKO
G
WT
Rptor ECKO
% TCRβ+
CD4+
CD8+
H
TCRβ+ (% CD45+)
WT
Rptor ECKO
I
WT
Rptor ECKO
% TCRβ+
CD4+
CD8+
J
TCRβ+ (% CD45+)
WT
Rptor ECKO
K
WT
Rptor ECKO
% TCRβ+
CD4+
CD8+
L
TCRβ+ (% CD45+)
WT
Tsc2 ECKO
M
WT
Tsc2 ECKO
% TCRβ+
CD4+
CD8+

Supplemental Figure 8. Endothelial mTORC1 promotes BODIPY-C16 uptake in tumor-associated T cells. (A-C) WT (n=4) or *Rptor*^{ECKO} (n=5) male mice were inoculated with LLC tumors cells and injected with BODIPY-C16, as described in Figure 5. BODIPY-C16^{+} cells are shown as **(A)** a percent of CD8^{+} T cells, **(B)** median fluorescence intensity (MFI) in CD4^{+} T cells, or **(C)** percent of CD4^{+} T cells. Representative plots are shown. BODIPY-C16 MFI was normalized to WT controls, presented as fold change (FC). Unpaired t-test, BODIPY^{+} (% CD8^{+}), p=0.132; CD4^{+} MFI, p=4.33x10^{-3}; BODIPY^{+} (% CD4^{+}), p=0.030. **(D-E)** WT (n=6) or *Tsc2*^{ECKO} (n=6) female mice were inoculated with E0771-luc tumor cells and injected with BODIPY-C16, as in (A-C). Representative plots of BODIPY-C16 in **(D)** CD8^{+} T cells or **(E)** CD4^{+} T cells are shown. BODIPY-C16 median fluorescence intensity (MFI) was calculated and normalized to WT controls, presented as fold change (FC). Unpaired t-test; CD8^{+} T cells, p=0.0464; CD4^{+} T cells, p=0.0422. **(F-G)** WT (n=6) or *Rptor*^{ECKO} (n=6) female mice were inoculated with E0771-GFP-luc tumor cells. Metastatic tumors were analyzed by flow cytometry. **(F)** T cells (TCRβ^{+}) and **(G)** CD4^{+}/CD8^{+} T cells are shown as percentage (%) of CD45^{+} or TCRβ^{+}, respectively. Unpaired t-test: TCRβ^{+}, p=0.946; CD4^{+}, p=0.868; CD8^{+}, p=0.369. **(H-I)** WT (n=10) or *Rptor*^{ECKO} (n=5) female mice were inoculated with MMTV-PyMT-GFP-luc tumor cells. Metastatic tumors were analyzed for **(H)** T cells (TCRβ^{+}) and **(I)** CD4^{+}/CD8^{+} T cells, as described in (F-G). Unpaired t-test: TCRβ^{+}, p=0.275; CD4^{+}, p=0.960; CD8^{+}, p=0.932. **(J-K)** WT (n=7) or *Rptor*^{ECKO} (n=4) male mice were inoculated with LLC-GFP-luc tumor cells. Metastatic tumors were analyzed for **(J)** T cells (TCRβ^{+}) and **(K)** CD4^{+}/CD8^{+} T cells, as described above. Unpaired t-test: TCRβ^{+}, p=0.515; CD4^{+}, p=0.944; CD8^{+}, p=0.997. **(L-M)** WT (n=8) or *Tsc2*^{ECKO} (n=6) female mice were inoculated with E0771-GFP-luc tumor cells. Metastatic tumors were analyzed for **(L)** T cells (TCRβ^{+}) and **(M)** CD4^{+}/CD8^{+} T cells, as described above. Unpaired t-test: TCRβ^{+}, p=0.376; CD4^{+}, p=0.958; CD8^{+}, p=0.396. *p<0.05, ***p<0.005. Related to Figure 5.

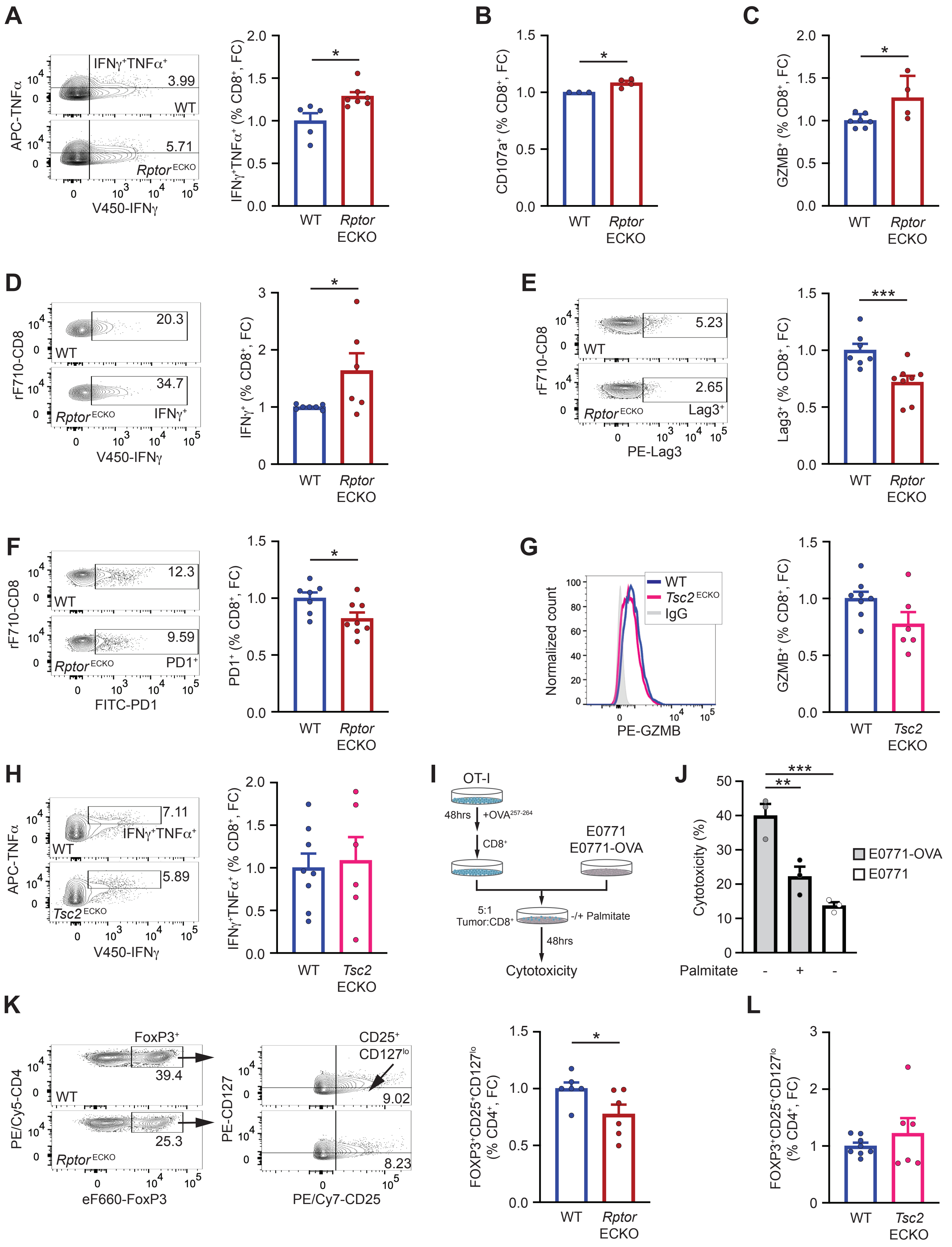

A
IFNγ+TNFα+
3.99
WT
5.71
Rptor ECKO
APC-TNFα
V450-IFNγ
IFNγ+TNFα+ (% CD8+, FC)
WT
Rptor ECKO
B
CD107a+ (% CD8+, FC)
C
GZMB+ (% CD8+, FC)
D
20.3
34.7
IFNγ+
rF710-CD8
IFNγ+ (% CD8+, FC)
E
5.23
2.65
Lag3+
PE-Lag3
Lag3+ (% CD8+, FC)
F
12.3
9.59
PD1+
FITC-PD1
PD1+ (% CD8+, FC)
G
WT
Tsc2 ECKO
IgG
Normalized count
PE-GZMB
Tsc2 ECKO
H
7.11
5.89
I
OT-I
48hrs
+OVA257-264
CD8+
E0771
E0771-OVA
5:1
Tumor:CD8+
-/+ Palmitate
48hrs
Cytotoxicity
J
Cytotoxicity (%)
E0771-OVA
E0771
Palmitate
K
FoxP3+
39.4
25.3
CD25+
CD127lo
9.02
8.23
PE/Cy5-CD4
eF660-FoxP3
PE-CD127
PE/Cy7-CD25
FOXP3+CD25+CD127lo (% CD4+, FC)
L

Supplemental Figure 9. Endothelial Raptor/mTORC1 loss improves anti-tumor immune activity of CD8$^+$ T cells. (A-B) WT or *Rptor*^{ECKO} female mice were inoculated with E0771-luc cells and lung metastatic tumors were analyzed by flow cytometry, as described in Figure 5. **(A)** TNFα^+IFNγ^+ and **(B)** CD107a$^+$ CD8$^+$ T cells were normalized to WT controls and shown as fold change (FC). TNFα^+IFNγ^+: WT (n=5), *Rptor*^{ECKO} (n=7); CD107a$^+$: WT (n=3), *Rptor*^{ECKO} (n=4). Unpaired t-test; TNFα^+IFNγ^+, p=0.0128; CD107$^+$, p=0.0144. **(C)** WT (n=7) or *Rptor*^{ECKO} (n=4) female mice were inoculated with MMTV-PyMT cells and lung metastatic tumors were analyzed by flow cytometry. CD8$^+$GZMB$^+$ cells were normalized, as described in (A). Unpaired t-test, p=0.0274. **(D)** WT (n=7) or *Rptor*^{ECKO} (n=6) male mice were inoculated with LLC tumor cells and lung metastatic tumors were analyzed by flow cytometry. CD8$^+$IFNγ^+ cells were normalized as in (A). Unpaired t-test, p=0.0473. **(E-F)** Metastatic tumor samples from (A) were analyzed for **(E)** Lag3 or **(F)** PD1. WT (n=7), *Rptor*^{ECKO} (n=8). Unpaired t-test; Lag3$^+$, p=0.00415; PD1$^+$, p=0.0303. **(G-H)** WT (n=8) or *Tsc2*^{ECKO} (n=6) female mice were inoculated and analyzed for **(G)** GZMB or **(H)** IFNγ/TNFα, as described in (A). Unpaired t-test; GZMB$^+$, p=0.0709; IFNγ^+TNFα^+, p=0.781. **(I-J)** The effects of palmitate on T cell cytotoxicity was determined. **(I)** Schematic of co-culture cytotoxicity assay. OT-I splenocytes were activated with OVA$^{257\text{-}264}$ (SIINFEKL) peptide for 48 hrs and positively selected for CD8. CD8$^+$ OT-I T cells were co-cultured with E0771-OVA cells in the absence or presence of 50 μM palmitate for 48 hrs, in a 5:1 tumor cell to T cell ratio. **(J)** Percent cytotoxicity was determined by LDH release (n=3 per group). E0771 parental cells do not express ovalbumin and were used as an antigen-free background control. One-way ANOVA (p=0.00126) with Tukey's post hoc. **(K)** WT (n=6) or *Rptor*^{ECKO} (n=6) female mice were inoculated with E0771 tumor cells, and lung metastatic tumors were analyzed by flow cytometry, as described in Figure 5. CD4$^+$FoxP3$^+$ Tregs were assessed for the activation markers CD25$^+$CD127^{lo}. Activated Tregs were normalized to WT controls and shown as fold change (FC). Unpaired t-test, p=0.0443. **(L)** WT (n=8) or *Tsc2*^{ECKO} (n=6) female mice were inoculated and analyzed as in (I). Unpaired t-test, p=0.375. *p<0.05, ***p<0.005. Related to Figure 5.

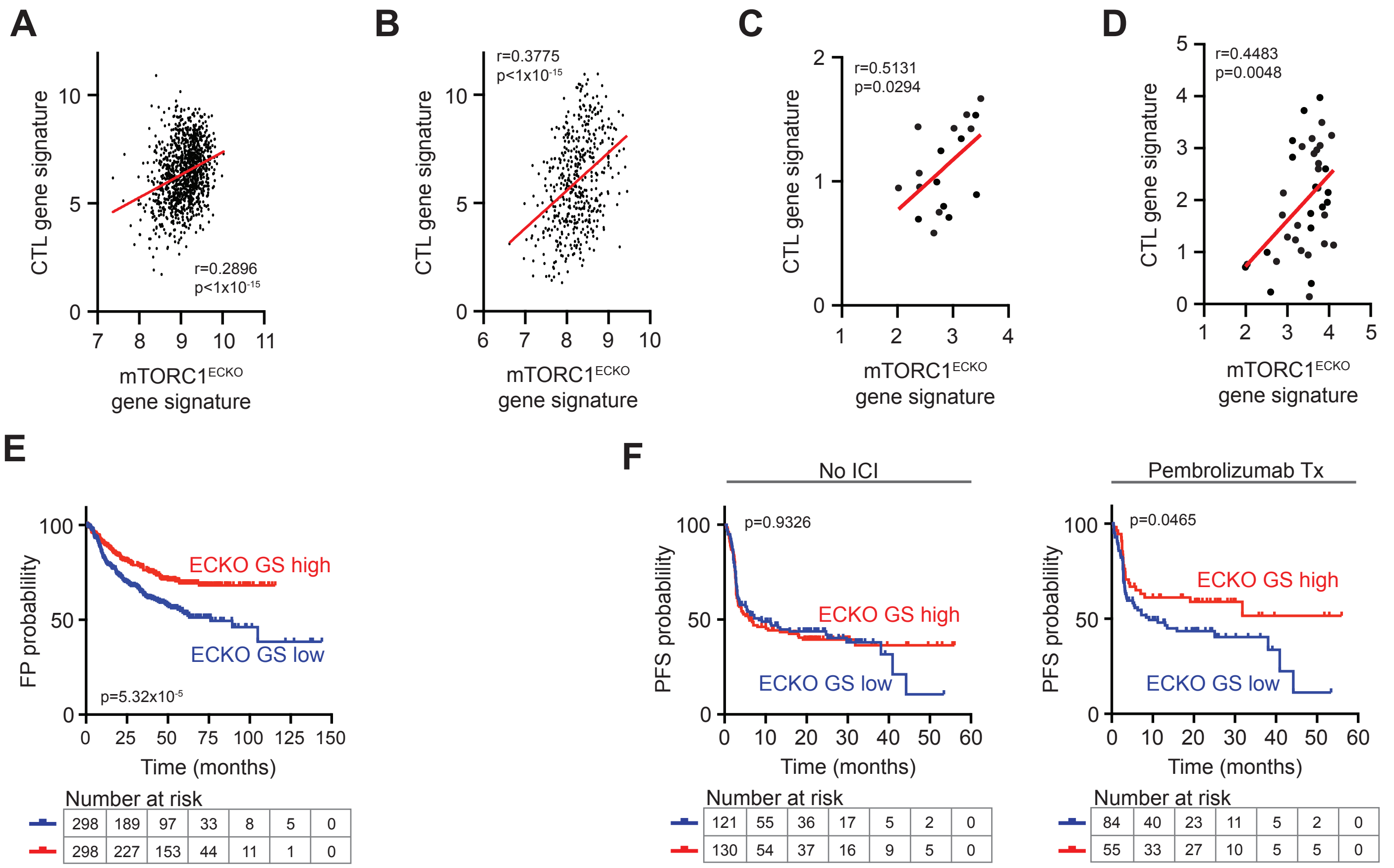

Supplemental Figure 10. Human tumors with a high mTORC1^{ECKO} gene signature correlate with improved anti-tumor T cell activity and improved progression-free survival. Differentially expressed genes in *Rptor*^{ECKO} tumor cells identified in Supplemental Figure 7G were used to generate an mTORC1^{ECKO} gene signature. **(A-D)** Correlations between the mTORC1^{ECKO} gene signature and a cytotoxic lymphocyte (CTL) gene signature in **(A)** TCGA LUNG (n=1129), **(B)** TCGA SKCM (n=474), **(C)** the Metastatic Breast Cancer Project (n=18), and **(D)** Metastatic Melanoma (n=38) datasets. **(E)** First progression (FP) survival of lung cancer patients, stratified by low (blue) or high (red) mTORC1^{ECKO} gene signature (ECKO GS). Number of at-risk patients in each group is shown. Hazard ratio (HR) is 0.5726 (95% CI: 0.4371 - 0.7502). **(F)** Progression-free survival (PFS) of melanoma patients treated with no immune checkpoint inhibitors (ICI, left) or pembrolizumab therapy (right). Patients were stratified by low (blue) or high (red) mTORC1^{ECKO} gene signature (ECKO GS). Number of at-risk patients in each group is shown. Hazard ratios (HR) for No ICI and Pembrolizumab therapy is 1.014 (95% CI: 0.7353 - 1.398) and 0.6101 (95% CI: 0.3839 - 0.9697), respectively. Related to Figure 5 and Supplemental Table 5.

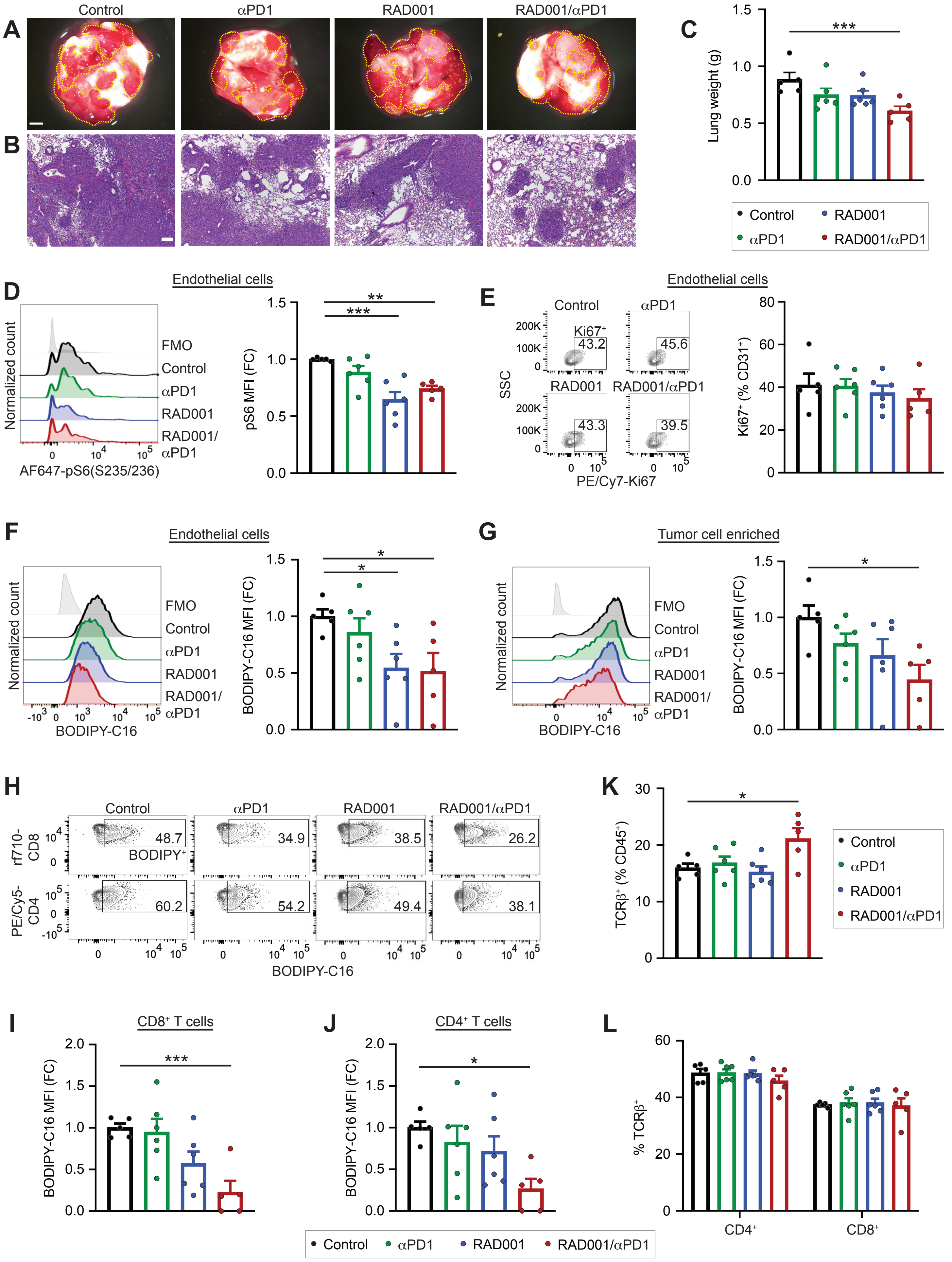

A
Control
αPD1
RAD001
RAD001/αPD1
B
C
Lung weight (g)
***
Control
αPD1
RAD001
RAD001/αPD1
D
Endothelial cells
Normalized count
FMO
Control
αPD1
RAD001
RAD001/αPD1
AF647-pS6(S235/236)
pS6 MFI (FC)
**
***
E
Endothelial cells
Control
αPD1
RAD001
RAD001/αPD1
Ki67+
43.2
45.6
43.3
39.5
SSC
PE/Cy7-Ki67
Ki67+ (% CD31+)
F
Endothelial cells
Normalized count
BODIPY-C16
BODIPY-C16 MFI (FC)
G
Tumor cell enriched
Normalized count
BODIPY-C16
BODIPY-C16 MFI (FC)
H
Control
αPD1
RAD001
RAD001/αPD1
rf710-CD8
PE/Cy5-CD4
BODIPY+
48.7
34.9
38.5
26.2
60.2
54.2
49.4
38.1
BODIPY-C16
K
TCRβ+ (% CD45+)
*
I
CD8+ T cells
BODIPY-C16 MFI (FC)
***
J
CD4+ T cells
BODIPY-C16 MFI (FC)
*
L
% TCRβ+
CD4+
CD8+

Supplemental Figure 11. Low-dose RAD001 combined with anti-PD1 reduces intratumoral BODIPY-C16 transport in lung metastatic tumors. Female wild type mice were inoculated with E0771-luc cells, as described in Figure 5. Control (n=5), αPD-1 (n=6), RAD001 (n=6), RAD001/αPD-1 (n=5). **(A)** Representative lung images show reduced surface tumors in RAD001/αPD1 animals. Scale bar is 5 mm. Visible tumor area is outlined by the yellow line. **(B)** Representative images H&E staining indicates reduced micro-metastatic tumor burden in RAD001/αPD1 lungs. Scale bar is 100 μm. **(C)** Lung weights were recorded at harvest in grams (g). One-way ANOVA (p=0.0167) with Dunnett's post hoc. **(D-E)** Within the $CD45^-CD31^+$ endothelial cell population, **(D)** phospho-S6 (S235/236) median fluorescence intensity (MFI) and **(E)** percent $Ki67^+$ cells was determined by flow cytometry. pS6 MFI was normalized to Control. One-way ANOVA (pS6, $p=4.26x10^{-4}$; Ki67, p=0.688) with Dunnett's post hoc where appropriate. **(F-G)** BODIPY-C16 median fluorescence intensity (MFI) was determined by flow cytometry in **(F)** $CD45^-CD31^+$ endothelial cells and **(G)** $CD45^-CD31^-FSC^{hi}$ tumor-cell enriched populations and normalized to Control. Representative histograms are shown. One-way ANOVA ($CD31^+$, p=0.0372; tumor-cell enriched, p=0.0443) with Dunnett's post hoc. **(H-J)** BODIPY-C16 MFI was determined in **(I)** $CD8^+$ and **(J)** $CD4^+$ T cells, as described in (D-E). Representative plots are shown in **(H)**. One-way ANOVA ($CD8^+$, p=0.00327; $CD4^+$, p=0.0367) with Dunnett's post hoc. **(K-L)** T cell populations were determined from treated lung metastatic tumors. **(K)** T cells ($TCR\beta^+$) and **(L)** $CD4^+/CD8^+$ T cells are shown as percentage (%) of $CD45^+$ or $TCR\beta^+$, respectively. One-way ANOVA ($TCR\beta^+$, p=0.0182; $CD4^+$, p=0.459; $CD8^+$, p=0.959) with Dunnett's post hoc where appropriate. *p<0.05, **p<0.01, ***p<0.005. Related to Figure 5.

Table S2. Genotyping primers used in study.

Primer Name		Primer Sequence
iCdh5-Cre	Forward	5'-TCCTGATGGTGCCTATCCTC-3'
	Reverse	5'-CGAACCTGGTCGAAATCAGT-3'
Rptor	Forward	5'-CTCAGTAGTGGTATGTGCTCAG-3'
	Reverse	5'-GGGTACAGTATGTCAGCACAG-3'
Rictor	Forward	5'-GAAGTTATTCAGATGGCCCAGC-3'
	Reverse	5'-ACTGAATATGTTCATGGTTGTG-3'
Tsc2	Forward	5'-TGGCAGGACAGAGGGTCATCATGG-3'
	Reverse	5'-TTCAGAGTCACCTGGCAGGCTCG-3'

Table S3. Mouse flow cytometry antibodies used in study.

Antibody	Clone	Dilution	Company	Catalog #
APC/Cy7-CD45	30-F11	1:500	BD Biosciences	557659
PerCP/Cy5.5-TCRβ	H57-597	1:500	Tonbo/Cytek	65-5961
PE/Cy5-CD4	GK1.5	1:500	Tonbo/Cytek	55-0041
PE/Dazzle 594-CD4	GK1.5	1:500	Biolegend	100455
Red Fluor 710-CD8	53-6.7	1:400	Tonbo/Cytek	80-0081
PE-CD8	53-6.7	1:400	BD Biosciences	553033
V450-CD107a	1D4B	1:200	BD Biosciences	560648
PE-Lag3	C9B7W	1:400	eBioscience	12-2231-81
FITC-PD1	29F.1A12	1:100	Biolegend	135213
PE/Cy7-CD25	PC61.5	1:500	Tonbo/Cytek	60-0251
PE-CD127	A7R34	1:500	Tonbo/Cytek	50-1271
BV421-CD31	390	1:200	Biolegend	102423
PE/Cy7-EpCAM	G8.8	1:500	Biolegend	118215
Super Bright 600-CD36	HM36	1:500	eBioscience	63-0362-82
PE/Cy7-Ki67	B56	1:100	BD Biosciences	561283
PE-GZMB	NGZB	1:20	eBioscience	12-8898-82
V450-IFNγ	XMG1.2	1:100	Tonbo/Cytek	75-7311
APC-TNFα	MP6-XT22	1:50	eBioscience	17-7321-82
eFluor 660-FoxP3	FJK-16s	1:250	eBioscience	50-5773-82
PE-IgG2α,κ	eBR2a	1:20	eBioscience	12-4321-81
APC-IgG1,κ	eBRG1	1:50	eBioscience	17-4301-82
FABP3		1:100	Invitrogen	PA5-13461
pS6 (S235/236)		1:100	Cell Signaling Tech.	2211

Table S4. Quantitative RT-PCR primers used in study.

Primer Name		Primer Sequence	Source
Mm-*Clstn1*	Forward	5’-GATGCCGTGGTAGTGGATAAG-3’	
	Reverse	5’-CCTGGATGGTGAATGTGTAGTC-3’	
Mm-*Rptor*	Forward	5’-CCTCTGTCCATATACGACCT-3’	
	Reverse	5’-CTGTGCAGTGCAAACTGT-3’	
Mm-*Cd36*	Forward	5’-GATGAGCATAGGACATACTTAGATGTG-3’	Hagberg CE, et al. *Nature.* 464:917-921 (2010).
	Reverse	5’-CACCACTCCAATCCCAAGTAAG-3’	
Mm-*Fatp1*	Forward	5’-TCAATGTACCAGGAATTACAGAAGG-3’	Hagberg CE, et al. *Nature.* 464:917-921 (2010).
	Reverse	5’-GAGTGAGAAGTCGCCTGCAC-3’	
Mm-*Fatp3*	Forward	5’-CCTCGGTTTCTCAGGCTCCA-3’	Hagberg CE, et al. *Nature.* 464:917-921 (2010).
	Reverse	5’-CTGTACCGGGCAGGTGTGA-3’	
Mm-*Fatp4*	Forward	5’-GCAAGTCCCATCAGCAACTG-3’	Hagberg CE, et al. *Nature.* 464:917-921 (2010).
	Reverse	5’-GGGGGAAATCACAGCTTCTC-3’	
Mm-*Fabp3*	Forward	5’-TTCAGCTGGGAATAGAGTTCG-3’	Hagberg CE, et al. *Nature.* 464:917-921 (2010).
	Reverse	5’-CTGCACATGGATGAGTTTGC-3’	
Mm-*Fabp4*	Forward	5’-GATGGTGACAAGCTGGTGGT-3’	Hagberg CE, et al. *Nature.* 464:917-921 (2010).
	Reverse	5’-AATTTCCATCCAGGCCTCTT-3’	
Mm-*Fabp5*	Forward	5’-GGAAGATGGCGCCTGGTGGA-3’	Hagberg CE, et al. *Nature.* 464:917-921 (2010).
	Reverse	5’-CCGAGTACAGGTGACATTGT-3’	
Mm-*Actb*	Forward	5’-AGAGGGAAATCGTGCGTGAC-3’	Edwards DN, et al. *J Clin Invest.* 131:e140100 (2021).
	Reverse	5’-CAATAGTGATGACCTGGCCGT-3’	

Table S5. Gene sets used in correlation and survival analysis of bulk RNA datasets.

Gene Sets	mTORC1^{ECKO} Gene Signature		CTL Gene Signature	
	DEGs from sorted tumor cells		Modified from Edwards et al. 2021.	
Gene Lists	**Gene Name**	**Gene ID**	**Gene Name**	**Gene ID**
	LCN2	ENSG00000148346	*CD8A*	ENSG00000153563
	PADI1	ENSG00000142623	*GZMA*	ENSG00000145649
	LYPD5	ENSG00000159871	*GZMB*	ENSG00000100453
	ARRB1	ENSG00000137486	*GZMM*	ENSG00000197540
	AMPD3	ENSG00000133805	*PRF1*	ENSG00000180644
	FAT2	ENSG00000086570	*IFNG*	ENSG00000111537
	MXD1	ENSG00000059728		
	VEGFA	ENSG00000112715		
	CRYAB	ENSG00000109846		
	DUSP10	ENSG00000143507		
	C3AR1	ENSG00000171860		
	IER2	ENSG00000160888		
	PTGS2	ENSG00000073756		
	SCN8A	ENSG00000196876		
	SGK1	ENSG00000118515		
	ERRFI1	ENSG00000116285		
	GPR35	ENSG00000178623		
	BDNF	ENSG00000176697		
	RCAN1	ENSG00000159200		
	GPR39	ENSG00000183840		
	DUSP1	ENSG00000120129		
	SOCS3	ENSG00000184557		
	TSC22D1	ENSG00000102804		
	ST3GAL1	ENSG00000008513		
	FILIP1L	ENSG00000168386		
	BACH1	ENSG00000156273		
	DNAJB9	ENSG00000128590		
	ARRDC3	ENSG00000113369		
	RBPJ	ENSG00000168214		
	HSPA5	ENSG00000044574		
	BIN1	ENSG00000136717		
	ITGB2	ENSG00000160255		
	TEAD2	ENSG00000074219		
	CGREF1	ENSG00000138028		
	FGFR1	ENSG00000077782		
	RAP1GAP	ENSG00000076864		
	CD109	ENSG00000156535		
	MICAL1	ENSG00000135596		
	PAPLN	ENSG00000100767		
	ACSL6	ENSG00000164398		
	CD22	ENSG00000012124		
