# Supplementary figures and images for: Increased fatty acid delivery by tumor endothelium promotes metastatic outgrowth
Source: JCI Insight. 2025 Apr 8;10(9):e187531. doi: 10.1172/jci.insight.187531 (PMC12128997; doi:10.1172/jci.insight.187531)

Full unedited blots for  
Supplemental Figure 4A

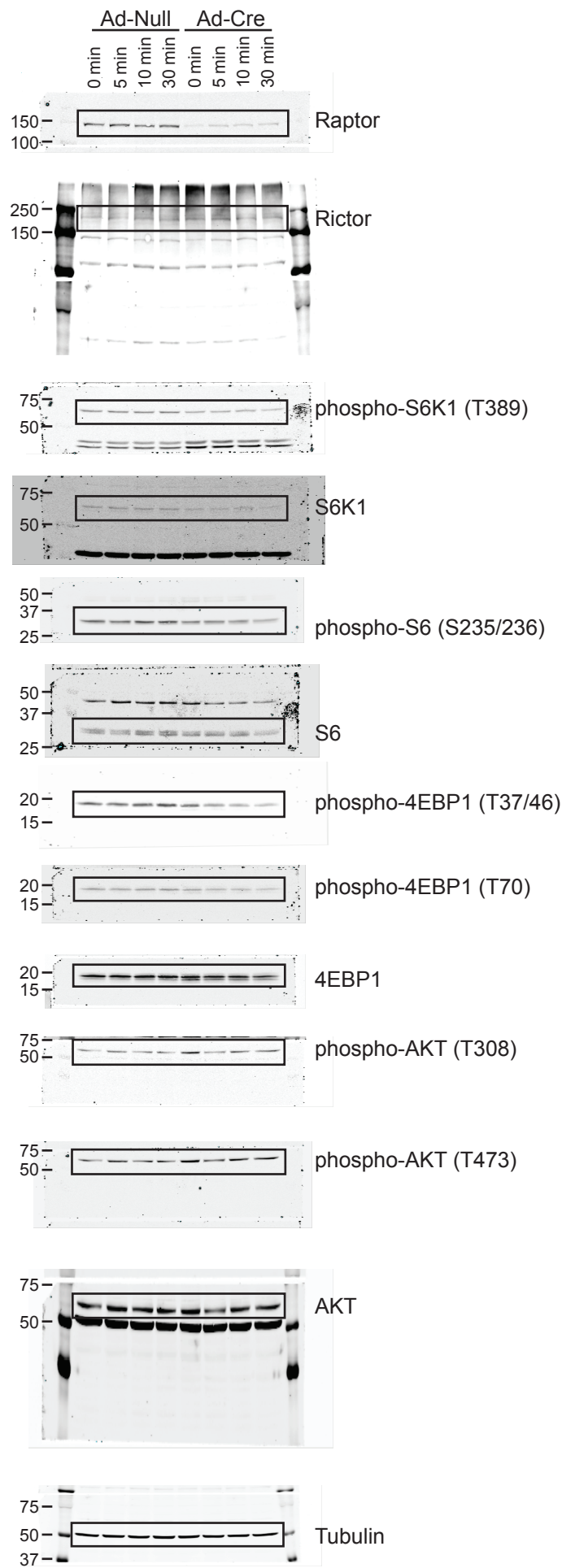

Full unedited blots for  
Supplemental Figure 5D

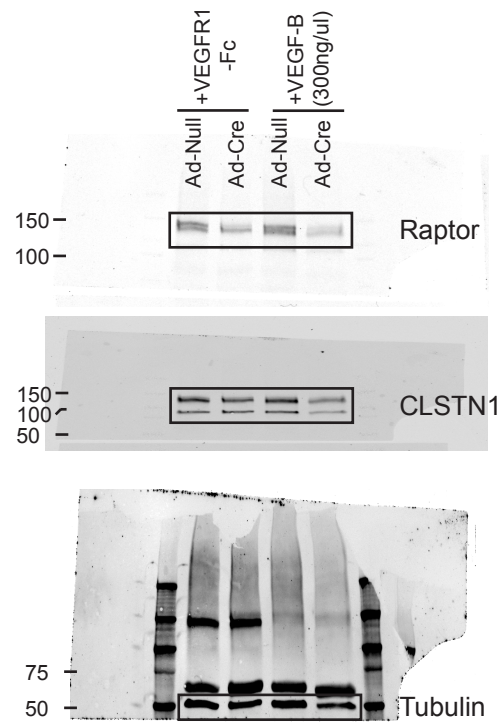

Supplement: Unedited blot and gel images [file jciinsight-10-187531-s069.pdf]
